# Histone lactylation boosted SET8 potentiates carcinogenesis and angiogenesis of pancreatic ductal adenocarcinoma by cooperating with MTA1/NuRD complex

**DOI:** 10.1186/s12967-026-08424-3

**Published:** 2026-06-26

**Authors:** Xujun Liu, Abulaihaiti Tuergong, Mingzhen Wang, Weilong Shi, Xiaodong Tian, Xia Ting, Liyan Cui, Wenzhe Si

**Affiliations:** 1 Department of Laboratory Medicine, State Key Laboratory of Vascular Homeostasis and Remodeling, Key Laboratory of Cardiovascular Molecular Biology and Regulatory Peptides of National Health Commission, Peking University Third Hospital, Beijing, 100191 China; 2 Department of Laboratory Medicine, Peking University First Hospital, Beijing, 100034 China; 3 Department of Pharmacy, Peking University Third Hospital, Beijing, 100191 China; 4 Department of General Surgery, Peking University Third Hospital, Beijing, 100191 China; 5 Department of Pathology, Peking University Third Hospital, Beijing, 100191 China; 6 Department of Laboratory Medicine, Peking University Third Hospital, Beijing, 100191 China

**Keywords:** Histone lactylation, SET8, MTA1/NuRD, Carcinogenesis, Angiogenesis

## Abstract

**Background:**

The pathophysiological role of lysine methyltransferase SET domain-containing protein 8 (SET8) in pancreatic ductal adenocarcinoma (PDAC) remains poorly understood. Emerging evidence suggests that histone lactylation, a novel post-translational modification, may influence tumor progression. However, the interplay between SET8 and lactate metabolism in PDAC pathogenesis has not been elucidated.

**Methods:**

Immunohistochemical staining and bioinformatics analysis of clinical databases were performed to evaluate SET8 expression and its prognostic relevance in PDAC. Affinity purification coupled with mass spectrometry (MS), co-immunoprecipitation (Co-IP), and glutathione S-transferase (GST) pull-down assays were conducted to identify interactions between SET8 and the MTA1/NuRD complex. Functional roles of the SET8/MTA1/NuRD complex were assessed using chromatin immunoprecipitation (ChIP)-seq, RT-qPCR, western blot, wound healing, transwell invasion, endothelial tube formation, in vivo Chicken Yolk Sac Membrane (YSM) assays, and Masson staining.

**Results:**

SET8 expression was significantly upregulated in PDAC and correlated with poor prognosis. SET8 physically interacted with the MTA1/NuRD complex via direct binding to MTA1, as confirmed by Co-IP and GST pull-down assays. The SET8/MTA1/NuRD complex repressed tumor suppressor genes, including SOCS2, through chromatin remodeling. Depletion of SET8 or MTA1 reduced tumorigenesis, angiogenesis, and metastasis. Overexpression of SET8 promoted oncogenic phenotypes in an MTA1-dependent manner. Mechanistically, lactate dehydrogenase A (LDHA)-mediated histone lactylation drove SET8 expression, linking metabolic reprogramming to epigenetic dysregulation.

**Conclusions:**

This study identifies SET8 as a proto-oncogene in PDAC, whose expression is regulated by histone lactylation. The SET8/MTA1/NuRD complex facilitates tumor progression by epigenetically silencing SOCS2, highlighting a crosstalk between histone methylation and deacetylation. Targeting the lactylation-SET8/MTA1/NuRD axis may offer a novel therapeutic strategy for PDAC, particularly by restoring SOCS2-mediated tumor suppression. These findings deepen our understanding of metabolic-epigenetic interplay in cancer and provide actionable insights for clinical intervention.

**Supplementary information:**

The online version contains supplementary material available at https://doi.org/10.1186/s12967-026-08424-3.

## Introduction

Pancreatic ductal adenocarcinoma (PDAC) is one of the most lethal human cancers with <5% overall 5 years survival rate [1, 2]. Despite decades of intensive research and effort, limited treatment options have been developed due to late diagnosis and chemoresistance for therapy of PDAC [3]. The dual blockade of angiogenesis and immune checkpoints is poised to become a standard of care in cancer treatment [4], and the development of effective anticancer drugs depends on fully elucidating the detailed tumorigenic mechanisms in cancers like PDAC.

Lactate is a product of glycolysis which has long been considered as a metabolic waste product. However, more and more studies indicate that lactate is a multifaceted molecule with diverse physiological and pathological functions [5], can result in the lactylation of histone lysine residues, thereby regulating gene transcription, metabolic reprogramming and immune evasion [6, 7]. Lactate dehydrogenase (LDH) catalyzes the conversion of pyruvate to lactate instead of acetyl-CoA, which is typically used as an intermediate in the tricarboxylic acid (TCA) cycle. LDH is a redox enzyme with a molecular weight of 134 kDa, which exists in five different isoforms (LDHA1–5) by various possible combinations of LDHA and LDHB subunits [8]. LDHA is a pivotal enzyme in glycolysis and is frequently overexpressed in PDAC [9]. Recently, we reported TTK protein kinase (TTK) activated LDHA, which upregulates lactate production and promoted histone lactylation [10]. However, the specific mechanisms responsible for the increased expression of LDHA and the elevation of lactylation in PDAC remain to be fully understood.

SET8 (also known as PR-Set7/9, SETD8, KMT5A) is a member of the SET domain-containing methyltransferase family, that specifically catalyzes the monomethylation of histone H4 at the lysine 20 residue (H4K20) [11]. SET8 has been implicated to play important roles in various biological processes, such as transcriptional regulation, maintenance of genome integrity, cell cycle progression and DNA damage response through its H4K20 specific methylase activity [12, 13]. Interestingly, SET8 has been reported to be involved in dual transcriptional activities both activation and repression [14] and revealed to function as an oncogene in several tumor types, including breast cancer [11], gastric carcinoma [15], hepatocellular carcinoma [16]. However, the involvement and mechanisms of SET8 in PDAC remains unknown.

The aim of the present study is to elucidate the mechanism by which SET8 affects the development of PDAC. Firstly, we reveal LDHA shows significant positive correlation with SET8, suggesting a potential association between SET8 and lactate metabolism. More evidences were provided which contribute to the increased SET8 expression by histone lactylation. The overexpression of SET8 coordinates with the MTA1/nucleosome remodeling and deacetylation (NuRD) complex, adding histone methylation activity to this complex, and implicating SET8/MTA1/NuRD in PDAC carcinogenesis and angiogenesis though inhibition SOCS2.

## Material and methods

### Human samples

PDAC tissue microarray (TMA) samples and their corresponding adjacent normal tissues were obtained from Outdo Biotech (Shanghai, China) which also provided patients’ information including gender, age, pathological grade and TNM stage. Use of the TMAs ethical approval was granted by the Clinical Research EthicsCommittee, Outdo Biotech Co., Ltd. The tissues of PDAC and paired para-carcinoma controls were archived from department of General Surgery at Peking University Third Hospital and approved by the Ethics Committee of Peking University Third Hospital.

### Cell lines and transfection

The PDAC cell lines MIA PaCa‑2 and PANC‑1 were purchased from the American Type Culture Collection (Manassas, VA, USA). AsPC-1 cells were obtained from the Cell Resource Center, Peking Union Medical College (Beijing, China), hTERT-HPNE cells were obtained from Meisen Cell Technology Co., Ltd (Zhejiang, China), the cells were cultured as previously described [10]. Primary human umbilical vein endothelial cells (HUVECs) were purchased from New Cell& Molecular Biotech Co., Ltd (Jiangsu, China) and maintained in Endothelial Cell Medium (Wuhan Procell Biotechology Co., Ltd.). Plasmids were prepared from Gene Chem (Shanghai, China) and lipofectamine 3000 (Invitrogen, CA, USA) was employed for transfection according to the manufacturer’s protocol. Lentivirus-based control shRNA and specific targeting shRNAs were purchased from Sigma-Aldrich. RNA interference was performed using Lipofectamine™ RNAi MAX (Invitrogen).

### Immunopurification and mass spectrometry

MIA Paca-2 cells stably expressing FLAG-SET8 were lysed in lysis buffer (50 mM Tris·HCl (pH 7.4), 150 mM NaCl, 0.3% Nonidet *P*-40, 1 mM DTT and 5 mM EDTA) containing protease inhibitor cocktail (Roche) and phosphatase inhibitor (Applygen, Beijing, China). After centrifugation at 15,000 g for 15 min at 4°C, the protein supernatant was incubated with anti-FLAG M2 immunoaffinity resin (Sigma-Aldrich) for 2 h at 4°C. FLAG peptide (0.2 mg/ml; Sigma-Aldrich) was applied to the resin to elute protein complex. The eluates were collected and resolved on NuPAGE 4–12% Bis-Tris gel (Invitrogen) and silver-stained. The bands were excised and subjected to liquid chromatography–tandem mass spectrometry sequencing and data analysis.

### Co-immunoprecipitation

For Co-IP assays, 500 μg of cellular extracts were incubated with indicated primary antibodies or normal rabbit/mouse immunoglobin G (IgG) (Immunoway) as negative controls on a rotator at 4°C overnight, 60 μl of 50% protein A or G agarose beads were added into to form immune complexes. After incubation for 2 h at 4°C with rotation, beads were washed five times with lysis buffer. The bound proteins were subjected to SDS-PAGE followed by IB with secondary antibodies.

### Glutathione S-transferase (GST) pull-down

GST fusion constructs were expressed in BL21 E. coli bacteria. The in vitro transcription and translation experiments were performed with rabbit reticulocyte lysate (TNT systems, Promega Biotech Co.,Ltd, Shanghai, China) according to the manufacturer’s recommendation. GST pull-down assay was performed as described previously [17].

### RT-qPCR

Total RNA was extracted using TRIzol reagent (Invitrogen). The RNA concentration was determined with a NanoDrop ND‑1000 spectrophotometer (NanoDrop Technologies; Thermo Scientific Fisher, Inc.) at 260 and 280 nm (A260/280). 2 μg of RNA in each group was reverse-transcribed (RT) into cDNA (i-presi, Beijing, China) and subjected to ABI 7500 PCR system (Applied Biosystems, Foster City, CA, USA) using SYBR Green I Kit (Roche Applied Science, Mannheim, Germany) according to the manufacturer’s protocol. All mRNA expression was standardized to GAPDH, and the mRNA levels were determined by the 2^‑ΔΔCq^ method. The primer sequences are listed in Supplementary Table S1.

### Western blot analysis

Total protein was extracted by lysis in RIPA buffer (Solarbio Life Sciences, China) containing protease inhibitor cocktail (Roche) for 15 min at 4˚C and centrifuged at 15,000 ×g for 15 min at 4˚C. The proteins concentration was measured by BCA assay (WB6501, New Cell & Molecular Biotech, China). Equal amounts of protein were subjected to 10–15% SDS‑PAGE and then transferred to NC membranes. The membranes were incubated sequentially with primary antibodies overnight at 4˚C and HRP‑linked secondary antibody for 1 h at room temperature, diluted with primary/secondary antibody dilution buffer (W003, applygen, China) and then visualized with a chemiluminescence substrate kit (Pierce™ ECL Western Blotting Substrate; Thermo Scientific Fisher, Inc.).

### Wound healing assay

The PDAC cells were seeded in 96-well plates and grown to 90% confluence, scratching wounds were generated by the IncuCyte Wound Marker tool. After washing with PBS, the culture medium was replenished with serum‑free medium, the culture was maintained for 48 h. Photographs were captured at intervals of 3 h using the IncuCyte S3 platform. The wound areas were quantified according to IncuCyte S3 image analysis software.

### Cell invasion assay

Boyden chambers (24‑well) (8‑μm pore size; Corning Inc., Corning, NY, USA) and Matrigel (BD Biosciences) was used in the invasion assays according to the manufacturer’s protocol. The cells were seeded at 2 × 10^5^ cells/well in the upper chamber with 500 μl serum‑free medium, while medium with 10% FBS was added to the lower chamber as a chemoattractant. After incubation for 48 h, cells that adhered to the lower surface were fixed in 4% paraformaldehyde (PFA) and then stained with 0.1% crystal violet. The numbers of stained cells were counted using an inverted microscope (Tokyo, Japan) in 5 random fields. All experiments were performed in triplicate.

### Tube formation assay

Angiogenesis was detected using HUVECs and basement membrane matrix (BD Biosciences) in vitro. 200 μl matrigel was coated on each well of the 24-well cell plates and was polymerized at 37°C. HUVECs (5 × 10^4^ cells/well) infected with the indicate viruses or cultured with CM from MIA Paca-2 cells that were infected with corresponding viruses. 6 h after incubation, the number of endothelial cell tubes was quantified and pictures were taken under a light microscope. WimTube quantitative tube formation image analysis was used to measure the cumulative tube length.

### Chicken yolk sac membrane (YSM) assay

Fertilized eggs were chosen and cracked on day 3 under sterile conditions. On day 9, gelatin sponges were cut to a size of 1 mm^3^ and placed on top of the YSM. The gelatin sponges were presoaked in MIA Paca-2 cell suspensions (approximately 2 × 10^5^ cells/sponge) which infected with viruses carrying the relative construct. On day 12, the vessels of the YSM were examined and counted under a light microscope.

### Chromatin immunoprecipitation (ChIP)-seq and qChip assays

ChIP was performed using an EZ‑ChIP™ ChIP kit (Merck Millipore, Darmstadt, Germany) according to the manufacturer’s protocol. 1 × 10^7^ cells were crosslinked with 37% formaldehyde. The chromatin was cleaved into fragments (200~500 bp) by ultrasound, after incubated with indicate antibodies or normal IgG as negative controls, the DNA was washed and recovered by phenol-chloroform extraction, followed by ethanol precipitation and then subjected to in-depth whole genome DNA sequencing (CapitalBio Corporation, Beijing, China). For qChIP analysis, qPCR was performed with SYBR Green I Kit. The levels of DNA were determined by the 2^‑ΔΔCq^ method.

### Animal studies

The use of nude mice was approved by the Animal Care and Use Committee of Peking University Health Science Center. The detailed procedures were performed as described previously [18]. Briefly, 200 μl of matrigel only (BD Bio-sciences) or matrigel that was mixed with MIA Paca-2 cells (2 × 10^5^ cells) that were infected with lentiviruses carry-ing control shRNA or SET8 shRNA or/and MTA1 shRNA were subcutane-ously injected into the left underarm BALB/c nude mice (*n* = 6). After injection, all mice were sacrificed and the Matrigel plugs were retrieved; then, the Matrigel plugs were fixed with 4% paraformal-dehyde and stained with Masson’s trichrome and H&E.

### Immunohistochemistry

The tissues from patients and nude mice were fixed with 4% paraformaldehyde (Biosharp, China) overnight and fixed in paraffin, 5-µm sections were prepared. Tissue sections were incubated with antibodies at 4°C overnight, followed by incubation with horseradish peroxidase conjugated secondary antibodies. The samples were photographed using an optical microscope (Olympus, Japan).

### Statistical analyses

Statistical analyses were performed using SPSS statistical software 18.0 (SPSS, Inc., Chicago, IL, USA). The data were expressed as mean±SD from at least three independent experiments. Difference between two groups was evaluated by Student’s t‑test, while multiple groups by one‑way analysis of variance with Student‑Newman‑Keuls (SNK) method for comparing each two groups, respectively. Kaplan‑Meier curves with a log rank test was used to examine the statistical analysis for overall survival time. *p < 0.05, **p < 0.01 indicate different degrees of statistical significance as noted in the figures.

## Results

### SET8 is upregulated in PDAC and indicates poor prognosis

**Fig. 1:**
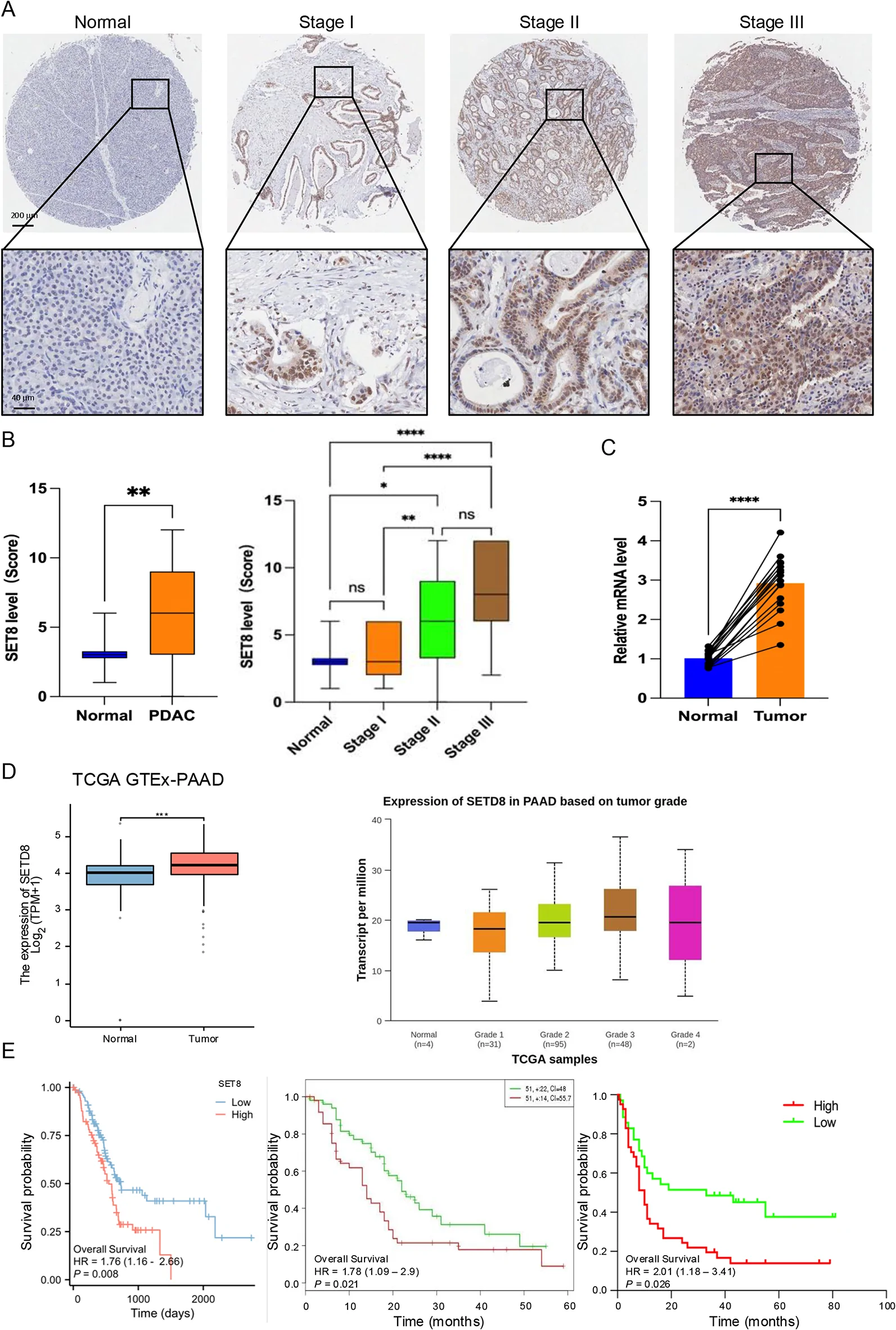

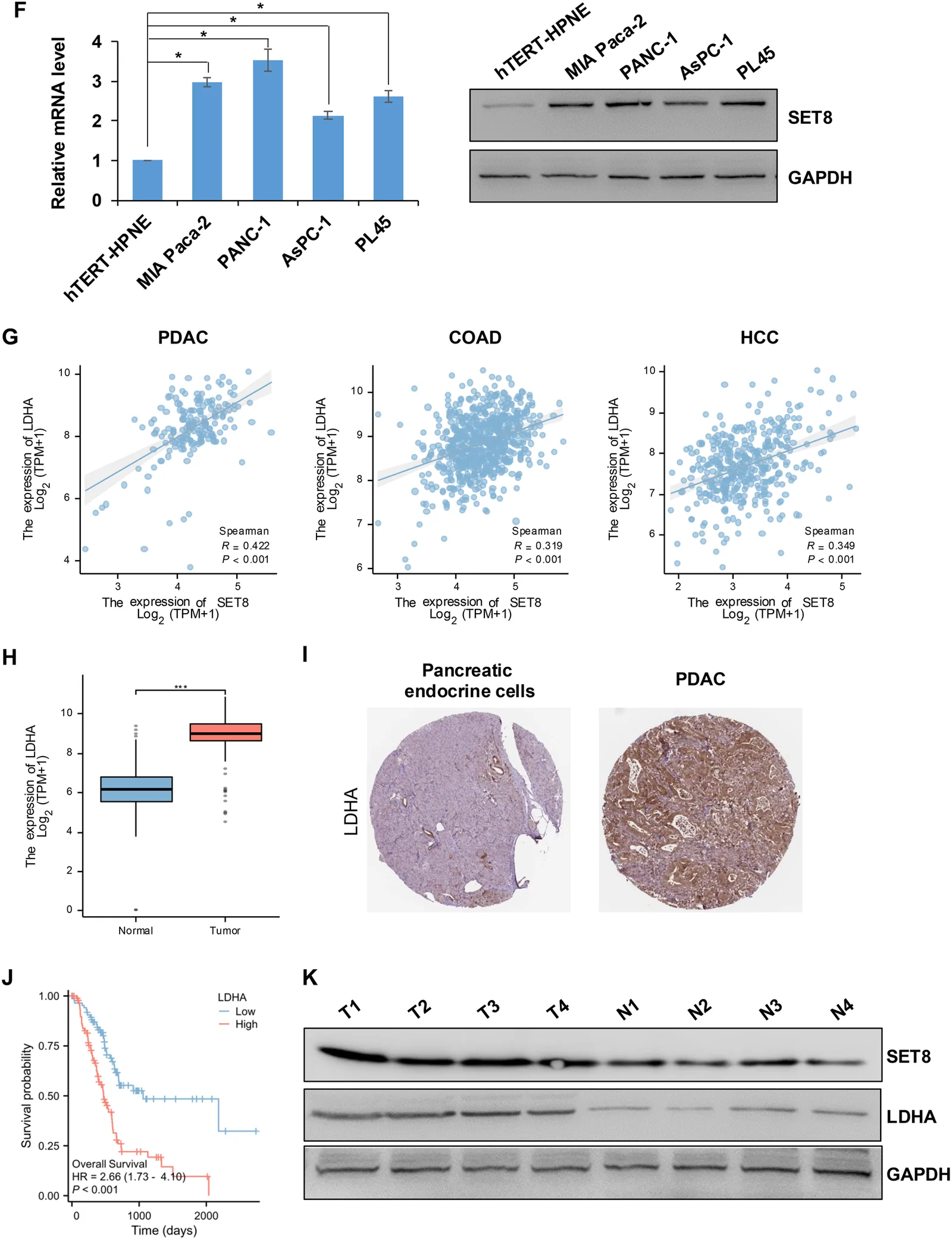
SET8 is upregulated in PDAC and indicates poor prognosis. (**A**) Immunohistochemistry staining images targeting SET8 (ab3798, 1:200, abcam) were obtained from PDAC specimens (*n* = 72) and adjacent para-carcinoma tissues (*n* = 74). Scale bars: up panel, 200 μm; Down panel, 40 μm. (**B**) Quantification of IHC staining, SET8 levels of across AJCC stages T1 to T4 in PDAC patients. T1 stage: 5 patients, T2 stage: 33 patients, T3 stage: 25 patients, T4 stage: 11 patients. (**C**) The mRNA expression levels of SET8 in 15 paired PDAC and adjacent tissue samples were examined using RT-qPCR. *p < 0.05. (**D**) Expression of SET8 was measured in PDAC tissue compared to paired non-tumor tissues in TCGA (GTEx-PAAD) and UALCAN datasets. (**E**) Kaplan-Meier survival curves for PDAC patients based on SET8 expression using TCGA_PAAD, GSE21501 database and TMA samples (*n* = 79). (**F**) Assessment of SET8 expression levels in human PDAC cell lines (MIA PaCa-2, PANC-1, AsPC-1, PL45) in comparison to the human pancreatic ductal epithelial cell line hTERT-HPNE. Data represented as mean ± SD for 3 experiments. *p < 0.05. (**G**) Correlation analysis of relative expression levels of SET8 and LDHA in PDAC, COAD and HCC within TCGA databases. (**H-I**) Expression of LDHA in PDAC tissues demonstrated by TCGA (GTEx-PAAD) dataset and the Human Protein Atlas. (**J**) Kaplan-Meier survival curves illustrating between PDAC patients with low LDHA levels and high LDHA levels. log rank test, *p* < 0.001. (**K**) Western blot analysis of SET8 and LDHA levels in PDAC tissues and adjacent para-carcinoma tissues

To elucidate the clinical significance and the mechanistic involvement of SET8 in human cancers, we conducted a comprehensive analysis of SET8 protein levels by immunohistochemical staining of human PDAC tissue microarray (TMA) samples, a statistically significant elevation of SET8 expression was observed in carcinomas compared to that in the para-carcinoma tissues, and its expression was positive correlated with more advanced American Joint Committee on Cancer (AJCC) stages (Fig. 1A–B). Furthermore, employing RT-qPCR on 15 out of 16 matched PDAC samples (one pair was excluded due to insufficient RNA quality) revealed an elevation in SET8 mRNA levels within tumor tissues compared to adjacent normal tissues (Fig. 1C). Notably, TCGA (GTEx-PAAD) database further confirmed the significantly elevated SET8 expression in PDAC tissues. The UALCAN database was searched (https://ualcan.path.uab.edu/index.html) [19] to further determine the expression of SET8 in PDAC. The expression level of SET8 were notably elevated in Grade 3 PDAC compared with Grade 1 (*p* < 0.05, Fig. 1D). Kaplan-Meier survival analysis was performed using data from TCGA_PAAD, the Gene Expression Omnibus (GSE21501), and TMA samples with follow-up data from Outdo Biotech. The analysis revealed that elevated SET8 expression was significantly associated with poorer overall survival in patients with PDAC (*p* < 0.05; Fig. 1E). Furthermore, SET8 expression positively correlated with larger tumor size and higher pathological grade (Supplementary Table S2). In addition, elevated SET8 levels were higher in the human PDAC cell lines MIA PaCa‑2, PANC‑1, AsPC-1 and PL45, in comparison to the human pancreatic ductal epithelial cell line hTERT-HPNE from mRNA and protein levels (Fig. 1F). Additionally, a significant positive correlation was observed between SET8 and lactate-producing enzyme LDHA in PDAC, colorectal adenocarcinoma (COAD), and hepatocellular carcinoma (HCC) (Fig. 1G), which indicates the potential association link between SET8 and lactate metabolism within digestive system malignancies. Representative results from the TCGA database confirmed that LDHA expression was increased in the PDAC tissues, providing further support for our hypothesis (Fig. 1H). Subsequently, we analyzed the LDHA protein level in clinical specimens from the online database of the Human Protein Atlas. LDHA displayed a positively strong expression in PDAC, contrasting with a weaker expression observed in adjacent normal pancreatic tissues (Fig. 1I). Furthermore, higher expression level of LDHA was associated with reduced overall survival times (Fig. 1J). Notably, LDHA and SET8 exhibited increased protein expression in paired PDAC tumors compared to the adjacent para-carcinoma tissues (Fig. 1K). Collectively, these observations suggest that the interplay between SET8 and LDHA might play an important role in PDAC progression.

### Histone lactylation potentiates the excessive expression of SET8

**Fig. 2:**
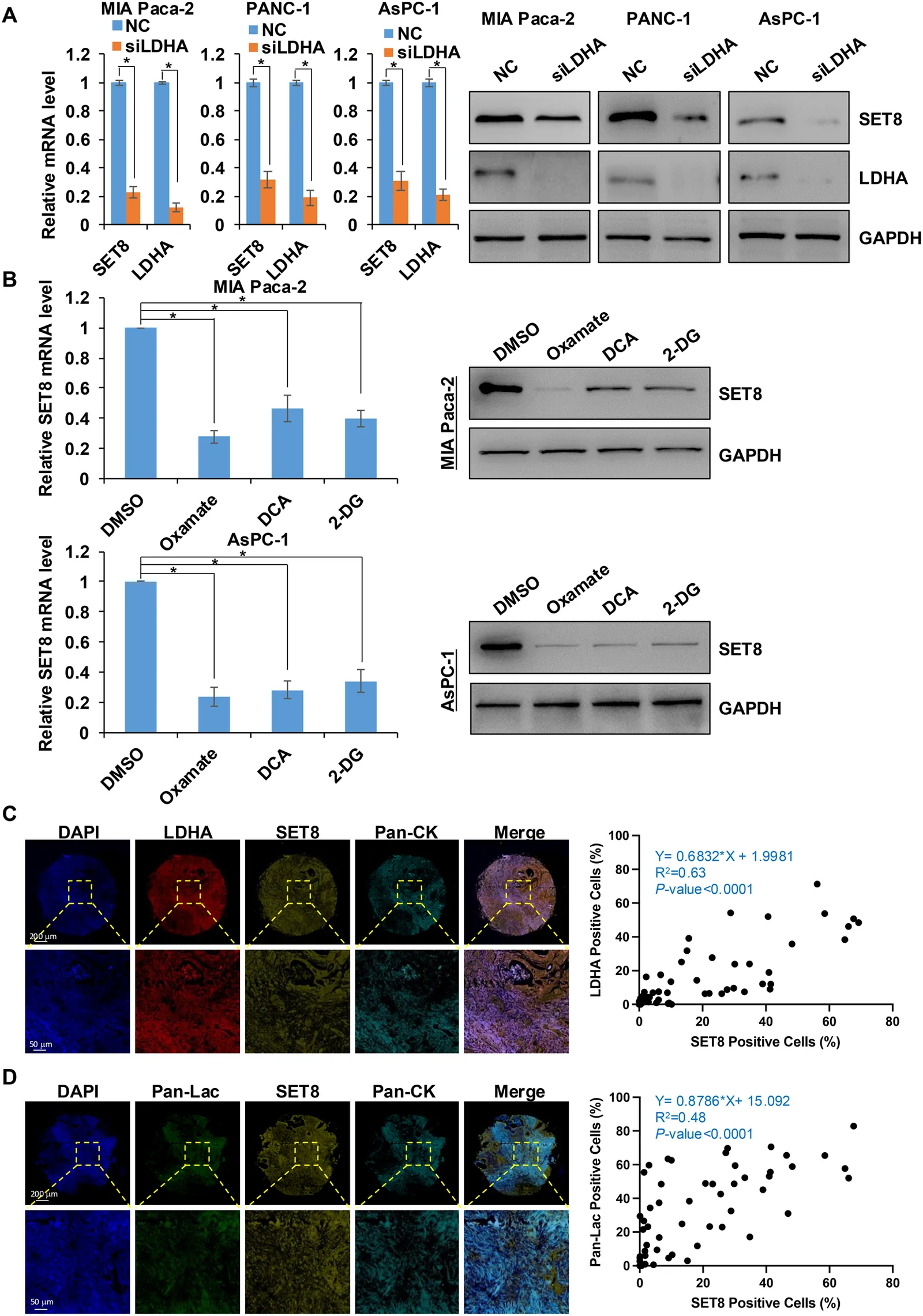

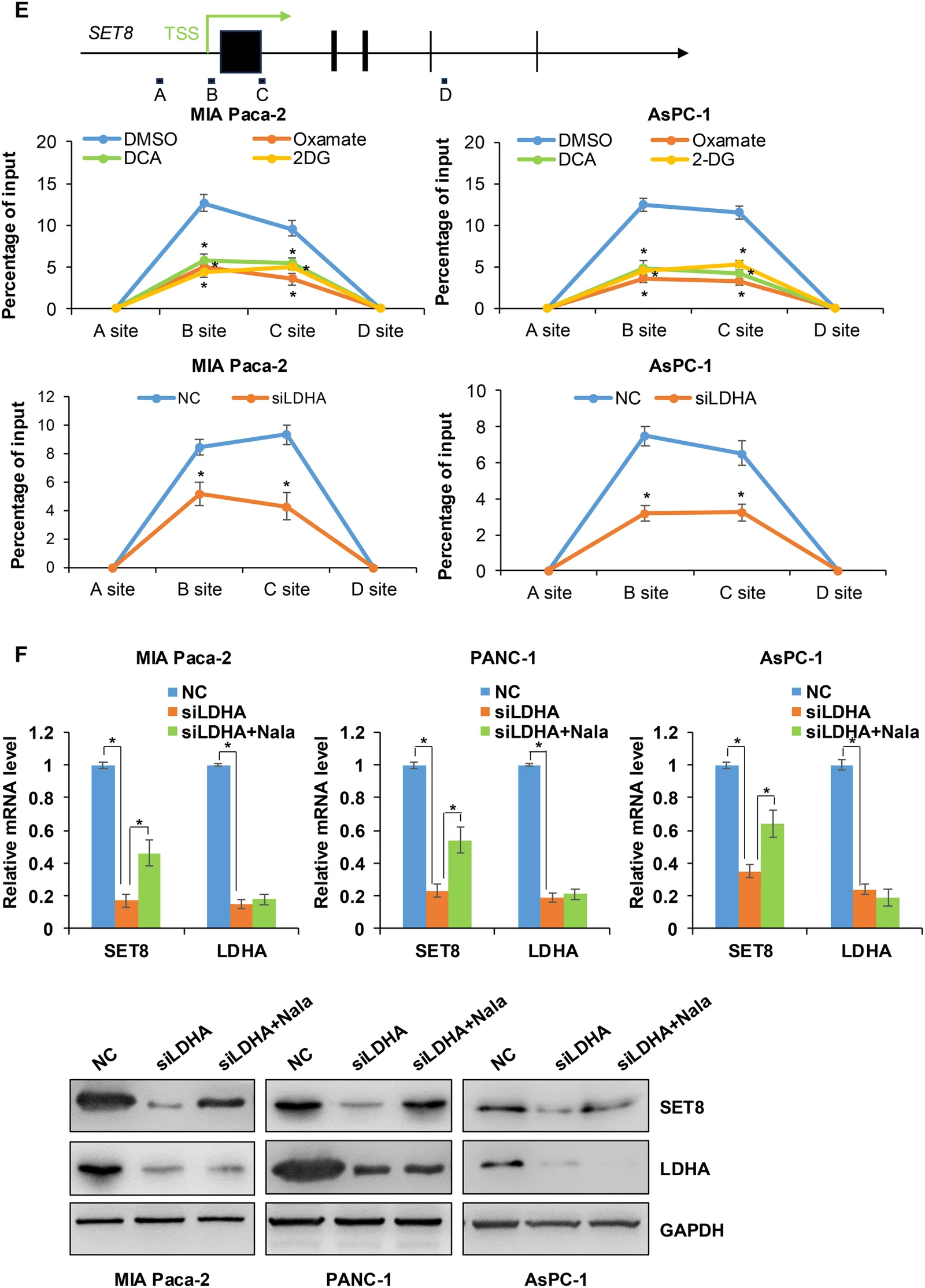
Histone lactylation potentiates the excessive expression of SET8. (**A**) RT-qPCR data and western blot demonstrating the expression levels of SET8 and LDHA in PDAC cells (MIA Paca-2, PANC-1 and AsPC-1) following LDHA knockdown. (**B**) Treatment of MIA Paca-2 and AsPC-1 cells with glycolysis inhibitors Oxamate (20 mmol/L), DCA (30 mmol/L), or 2-DG (10 mmol/L), followed by measurement of SET8 levels using RT-qPCR and western blot. (**C** and **D**) Immunofluorescence staining of SET8 (yellow), Pan-Lac (green), LDHA (red), Pan-CK (cyan) and DAPI (blue) in tumor samples. Scale bars: up panel, 200 μm; Down panel, 50 μm. Correlation analysis of the relative protein expression levels of SET8 and LDHA or Pan Lac in PDAC, assessed using Pearson correlation analysis. (**E**) ChIP-qPCR assay investigating the H3K18la status in the SET8 genomic region in PDAC cells (MIA Paca-2 and AsPC-1) upon treatment with glycolysis inhibitors (Oxamate, DCA, or 2-DG) or LDHA inhibition. Data presented as mean ± standard deviation of triplicate experiments. (**F**) Assessment of SET8 and LDHA expression via RT-qPCR and western blot analyses after LDHA knockdown and treatment with Nala

To determine the molecular basis underlying the increased expression of SET8 and LDHA, we first silenced LDHA expression in MIA Paca-2, PANC-1, AsPC-1 cell lines, the mRNA and protein expression of LDHA and SET8 were significantly reduced in the si-LDHA transfection groups (Fig. 2A). Furthermore, treatment with the LDHA inhibitor FX11 significantly reduced SET8 expression (Supplementary Figure S1A), supporting the association between LDHA activity and SET8 upregulation. Moreover, RT‑qPCR and western blot analysis confirmed that treatment with various glycolysis inhibitors, including DCA, Oxamate, and 2-DG significantly decreased SET8 levels in MIA PaCa-2 and AsPC-1 cells (Fig. 2B). Since our previous studies have indicated PDAC harbor increased lactylation and histone lactylation levels [10], a hypothesis was formulated suggesting a plausible association between the upregulation of SET8 and lactylation pathways. To test this hypothesis, we verified the expression pattern between lactylation and SET8 in PDAC TMA samples. Utilizing immunofluorescence techniques, we uncovered a pronounced positive correlation between SET8 and LDHA or lactylation protein expression (Fig. 2C–D). Treatment with glycolysis inhibitors or knockdown of LDHA resulted in dramatic decreased histone H3K18 lactylation levels in the promoter region of SET8 (Fig. 2E). In contrast, ChIP-qPCR analysis using an anti-H3K18la antibody confirmed significant enrichment at the SET8 promoter (B and C sites) in MIA PaCa-2 or AsPC-1 cells following 48-hour sodium lactate (Nala) treatment, a lactylation inducer (Supplementary Figure S1B). This provides direct evidence that lactylation regulates SET8 transcription. Furthermore, knockdown of LDHA followed by sodium lactate Nala treatment, a notable enhancement in SET8 levels was observed compared to the si-LDHA group across distinct PDAC cells respectively (Fig. 2F).

### SET8 directly interacts the MTA1/NuRD complex to coordinate histone methylation and deacetylation

**Fig. 3:**
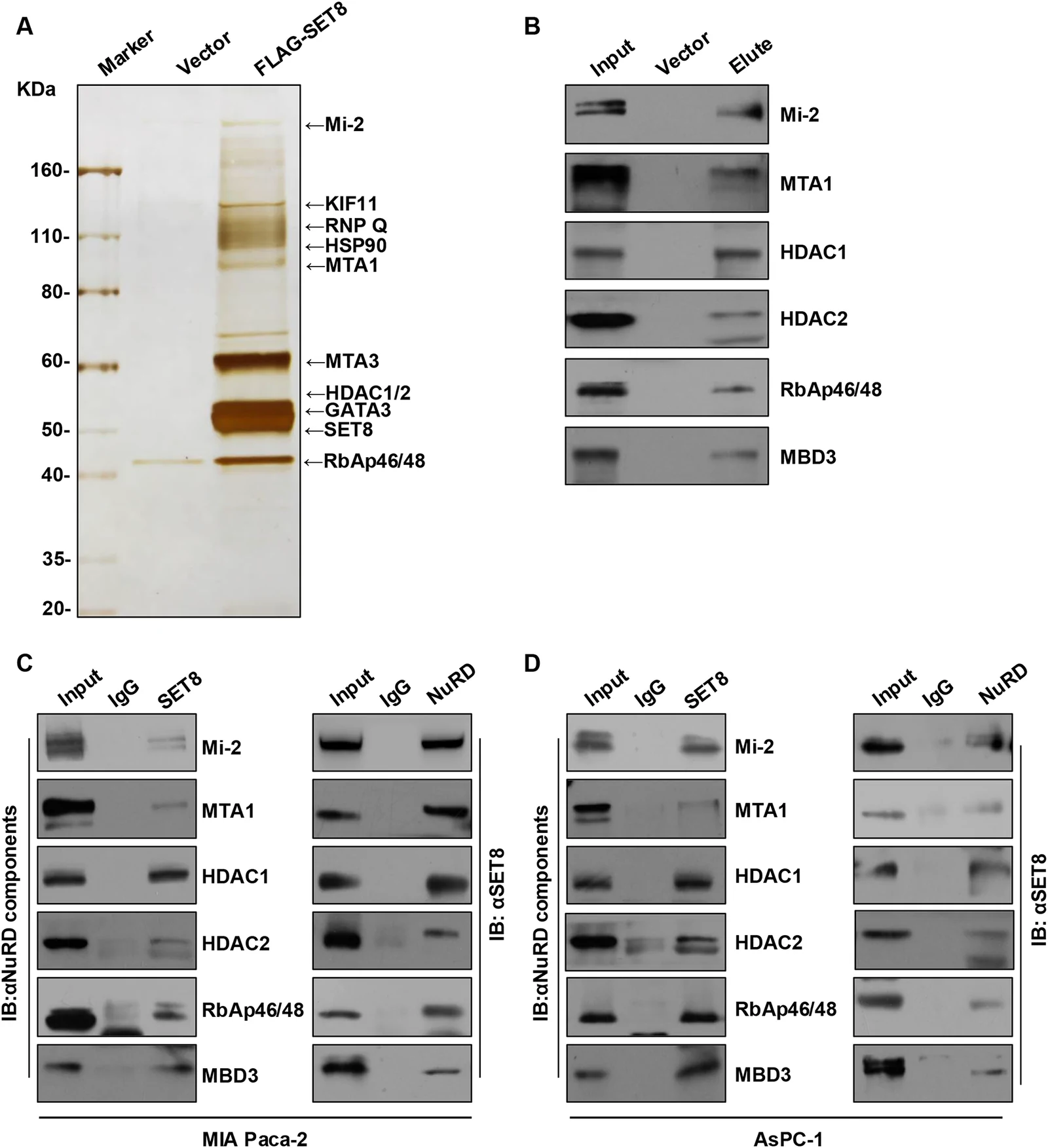

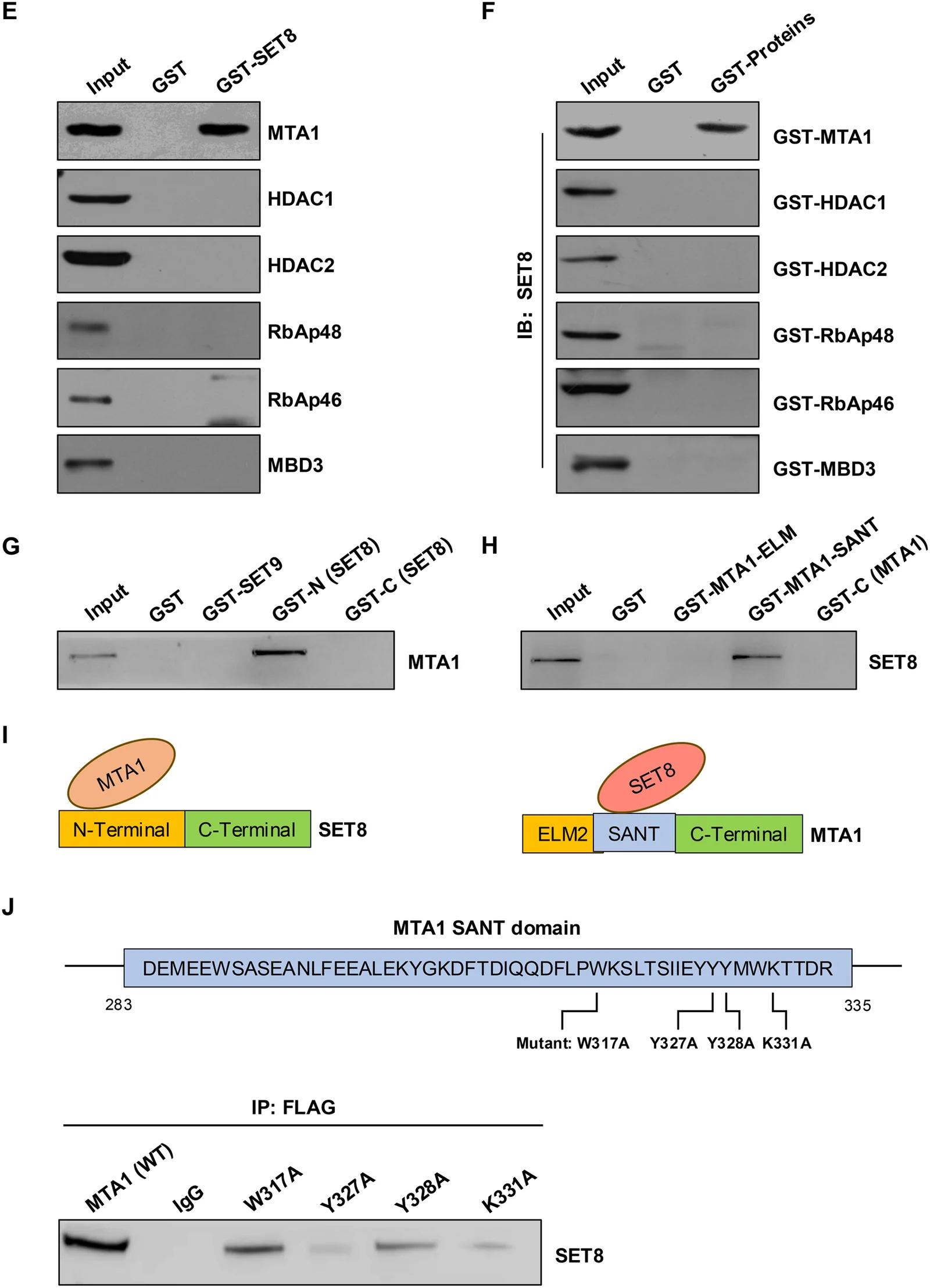

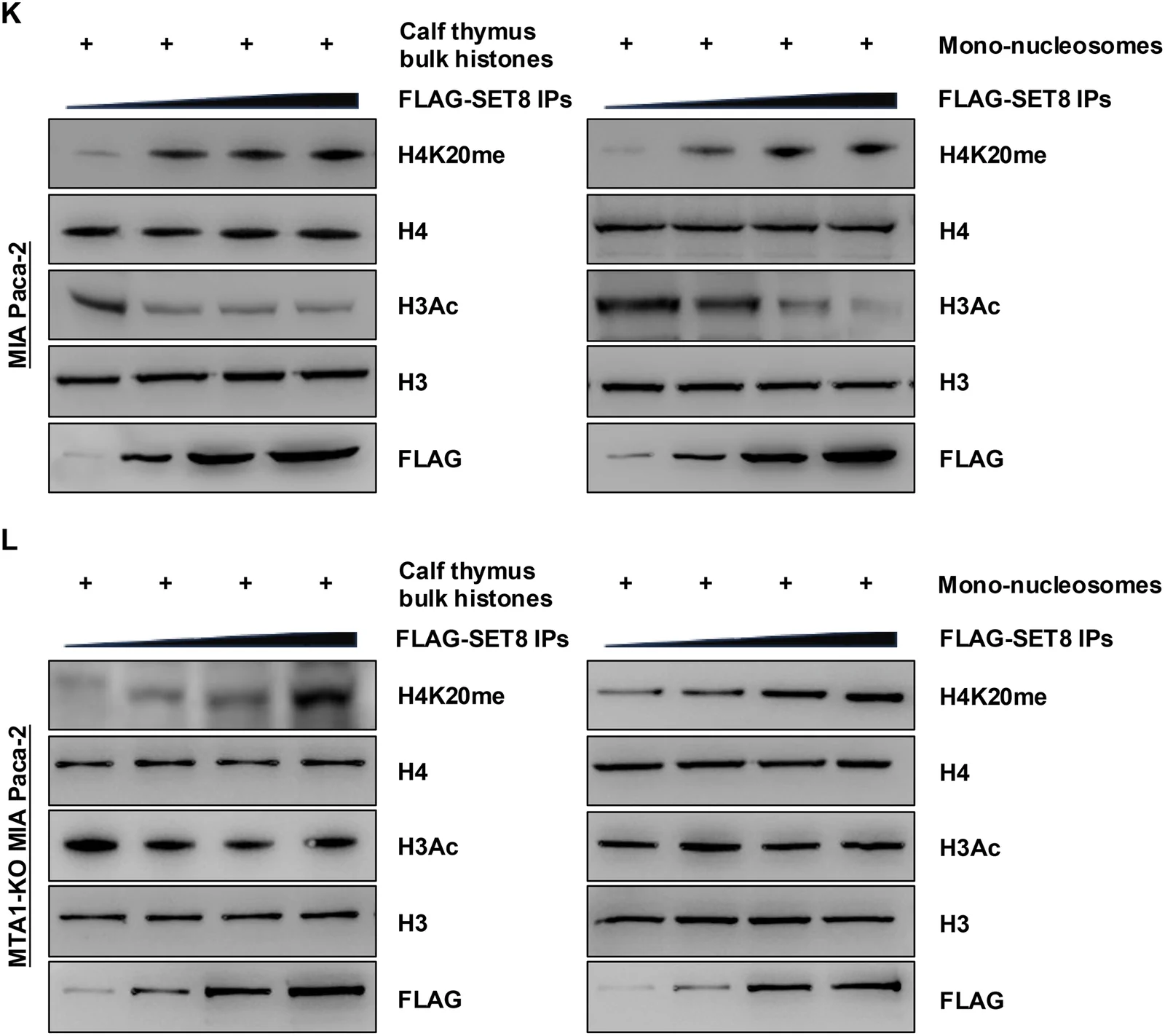
SET8 directly interacts the MTA1/NuRD complex to coordinate histone methylation and deacetylation. (**A**) Cellular extracts from MIA Paca-2 cells stably expressing FLAG-SET8 were immunopurified with anti-FLAG affinity columns, followed by elution with FLAG peptide. The eluted proteins were separated by SDS-PAGE and visualized by silver staining. Subsequently, the protein bands were retrieved and analyzed by mass spectrometry. MIA Paca-2 cells stably expressing FLAG-vector served as the negative control. (**B**) Western blot analysis was conducted to identify the proteins in the purified fractions, utilizing specific antibodies. Whole cell lysates from (**C**) MIA Paca-2 cells or (**D**) AsPC-1 cells were immunoprecipitated with antibodies against SET8 followed by immunoblotting with the antibodies against the MTA1/NuRD complex. Reciprocal association of MTA1/NuRD complex with the SET8 were analyzed. (**E-F**) GST pull-down analysis was carried out utilizing a combination of GST fusion proteins expressed in bacteria and proteins transcribed/translated in vitro. (**G**) Segmented GST pull-down analysis involved GST-fused SET9, SET8 N-terminal, SET8 C-terminal, as well as MTA1 proteins transcribed/translated in vitro. (**H**) GST-fused MTA1 ELM domain, SANT domain, C-terminal, and SET8 proteins transcribed/translated in vitro were used for GST pull-down analysis. (**I**) a schematic diagram illustrating the molecular interactions between SET8 and MTA1. (**J**) MIA Paca-2 Cells were tranfected with FLAG-MTA1, FLAG-MTA1 (W317A), FLAG-MTA1 (Y327A), FLAG-MTA1 (Y328A), FLAG-MTA1 (K331A) separately, and then immunoprecipitated with antibodies against FLAG followed by immunoblotting with the antibodies against SET8. (**K**) MIA Paca-2 cells or (**L**) MTA1-KO MIA Paca-2 cells, both overexpressing FLAG-SET8, were immunopurified on an anti-FLAG affinity column and eluted with FLAG peptides. These eluates were incubated with calf thymus histones or mononucleosomes isolated from HeLa cells in histone methylation or histone deacetylation assay buffer. The reaction was analyzed by western blotting with antibodies specific to the indicated histone marks or proteins

To better understand the mechanistic function of SET8, we employed affinity purification and mass spectrometry (MS) to investigate the physical interactome of SET8. Specifically, FLAG-SET8 was stably expressed in MIA Paca-2 cells, and cellular extracts were subjected to affinity purification using anti-FLAG affinity columns. The MS analysis showed that SET8 co-purified with CHD4 (Mi-2), MTA1, HDAC1/2, RbAp46/48, all components of NuRD complex, as well as several other proteins (Fig. 3A). The presence of the NuRD subunits within the SET8 interactome was accomplished through western blot of the column bound proteins with antibodies against the putative partner proteins (Fig. 3B). To further corroborate the interaction between SET8 and the NuRD complex, total proteins from MIA Paca-2 cells were extracted, and immunoprecipitation (IP) was performed with antibodies against SET8 followed by immunoblotting (IB) with antibodies against subunit of the NuRD complex, the results demonstrated that each of these tested NuRD components efficiently co-immunoprecipitated with SET8. Reciprocally, IP with antibodies against components of the NuRD complex and IB with antibodies against SET8 also revealed that SET8 was efficiently co-immunoprecipitated with the NuRD complex (Fig. 3C). Furthermore, consistent results were observed in AsPC-1 cells (Fig. 3D). To understand the molecular interaction between SET8 and the NuRD complex, GST pull-down assays were conducted employing GST-fused SET8 (GST-SET8) and individual in vitro transcribed/translated components of the NuRD complex. These experiments revealed that SET8 could interact specifically with MTA1 among the tested NuRD complex components (Fig. 3E). Reciprocally, GST pull-down assays utilizing GST-fused NuRD subunit and SET8 synthesized through coupled transcription/translation in vitro showed that MTA1was able to interact with SET8 (Fig. 3F). Taken together, these data suggest that the association of SET8 with the NuRD complex is through interactions with MTA1. To further map the detailed interface of between SET8 and the NuRD complex, GST-tagged truncated forms of SET8 were employed. Notably, our investigations revealed that N-terminal fragment (1–202 aa) of SET8 but not C-terminal SET domain (216–343 aa) or SET9 is responsible for the interaction with NuRD (Fig. 3G). Moreover, the SANT domain of MTA1 was responsible for this interaction with SET8 (Fig. 3H). In conclusion, our comprehensive analysis elucidated the molecular details underlying the formation of the SET8/MTA1/NuRD complex (Fig. 3I). Furthermore, we generated mutations at various sites in the SANT domain using the QuikChange Lightning Site-Directed Mutagenesis Kit. As shown in Fig. 3J, the protein-protein interaction was markedly disrupted by the Y327A mutation in the IP-binding residues, while mutations at W317A, Y328A, or K331A only resulted in a partial reduction of the interaction. To further substantiate the functional significance of the physical interaction between SET8 and the NuRD complex, the SET8-containing complex were IP from MIA Paca-2 cells expressing FLAG-SET8 with anti-FLAG affinity columns. Subsequently, these IPs were incubated with either calf thymus bulk histones or mono-nucleosomes isolated from HeLa cells, and the histone modifications were assessed via western blot analysis. As expected, the SET8-containing complex demonstrated a notable reduction in the acetylation level of H3, indicative of the enzymatic activity attributed to the NuRD complex (Fig. 3K). Moreover, an anti-FLAG antibody was utilized to precipitate SET8-containing protein complex from MTA1-knockout (KO) MIA Paca-2 cells expressing FLAG-tagged SET8. Analysis of these IPs, following incubation with calf thymus histones or mono-nucleosomes isolated from HeLa cells, by western blot with anti-H3ac antibody revealed markedly impaired interdependence of such activities (Fig. 3L). These experiments demonstrate that the interaction between SET8 and MTA1/NuRD complex is essential for the intricate crosstalk between histone methylation and deacetylation.

### Genome wide transcriptional targets for SET8/NuRD (MTA1) complex

**Fig. 4:**
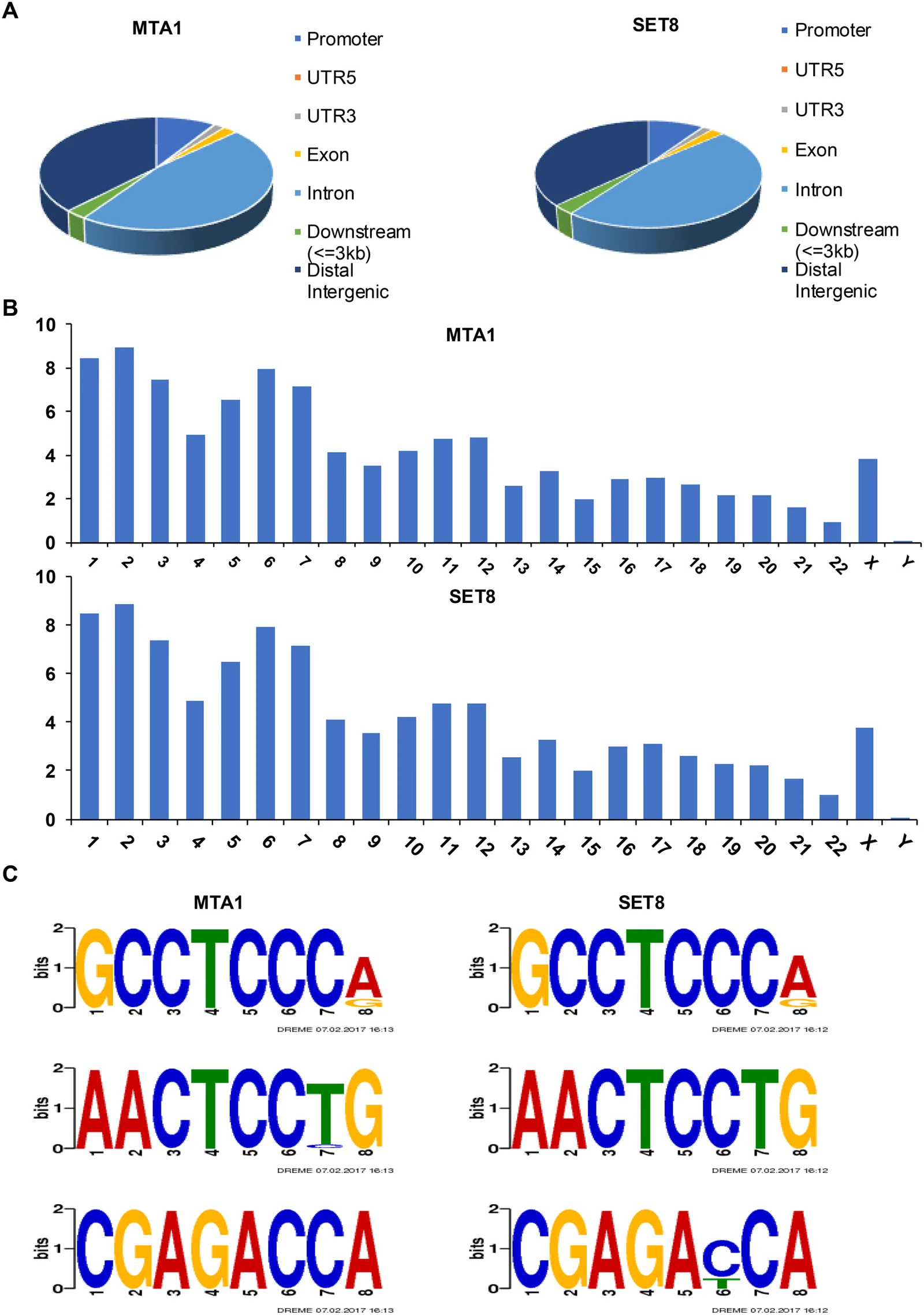

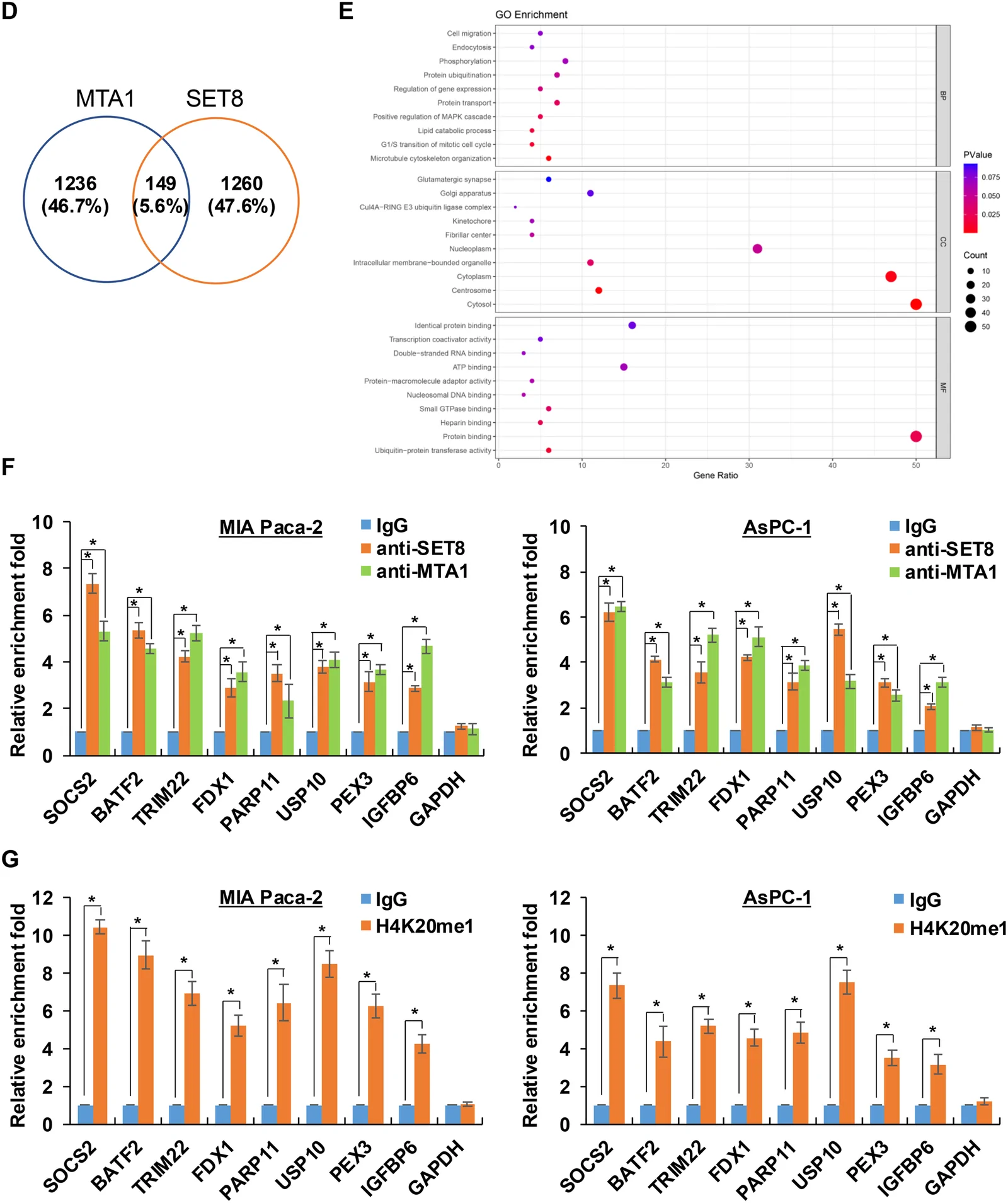

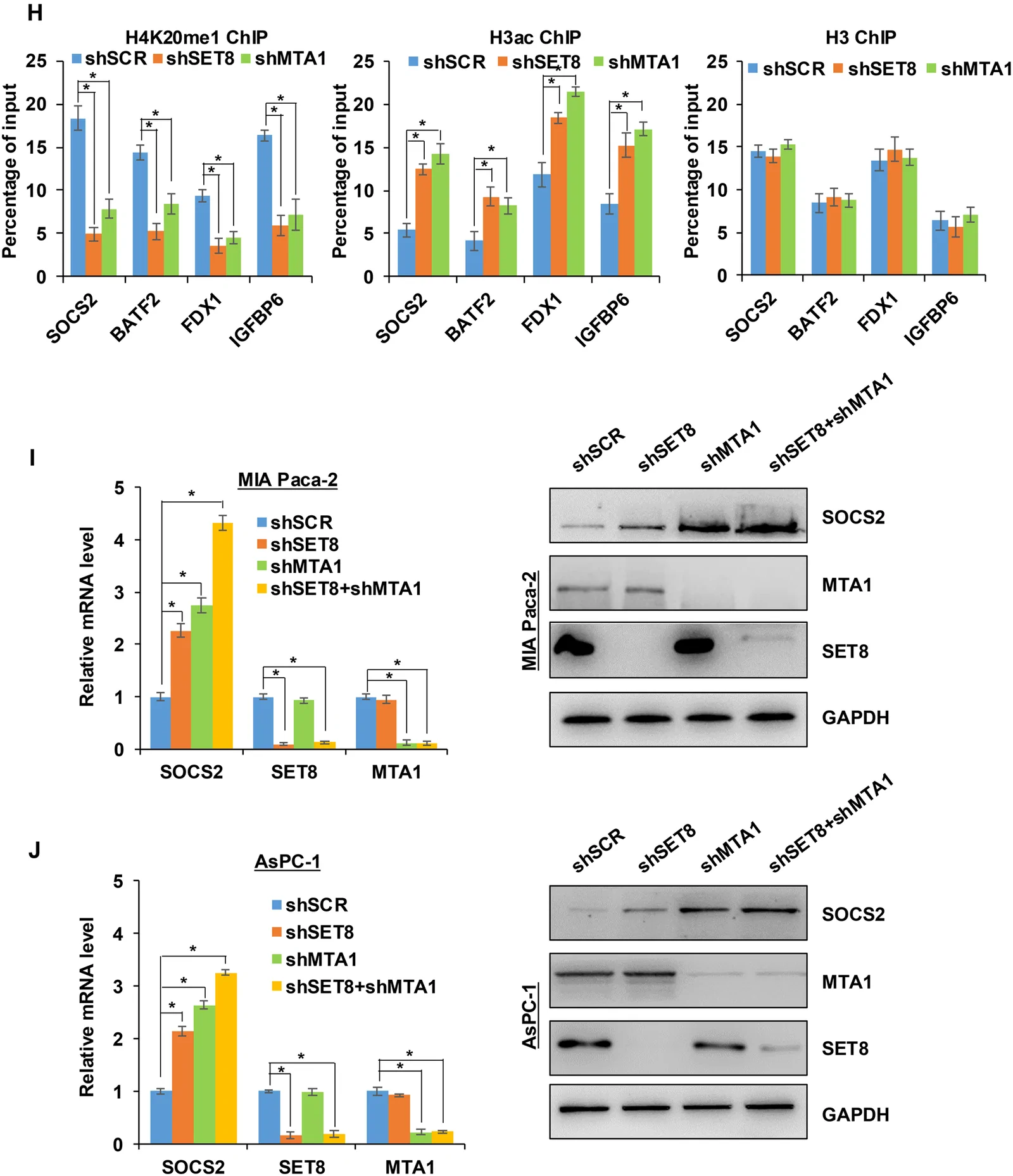
Genome wide transcriptional targets for SET8/NuRD (MTA1) complex. (**A**) ChIP-seq analysis was conducted to investigate the genomic distribution of SET8 and MTA1 in MIA Paca-2 cells and the peaks’ distribution is shown. (**B**) The chromosome distribution of the identified peaks is presented. (**C**) The bound motifs for SET8 and MTA1 were analyzed using MEME suite. (**D**) An overlapped promoter analysis was illustrated using a Venn diagram to show the common promoters bound by both SET8 and MTA1 in MIA Paca-2 cells. (**E**) The clustering of the overlapping target genes of SET8/MTA1 into GO enrichment analysis is displayed. (**F-G**) Verification of the ChIP-seq results by qChip analysis of the indicated genes used antibodies against SET8, MTA1, or H4K20me1 in MIA Paca-2 cells or AsPC-1 cells. Data is represented as Fold of change over control, with purified rabbit IgG serving as an internal reference control. GAPDH was used as a negative control. Error bars represent as mean ± SD for three independent experiments. (**H**) The recruitment on SOCS2, BATF2, FDX1 and IGFBP6 promoters for the indicated proteins including SET8, MTA1, H4K20me1, H3Ac and H3, was evaluated in MIA Paca-2 cells or AsPC-1 cells infected with knockdown lentiviruses (shSCR, shSET8, shMTA1) by qChip analysis. The expression levels of SOCS2, MTA1 and SET8 were assessed in (**I**) MIA Paca-2 cells and (**J**) AsPC-1 cells infected with knockdown lentiviruses (shSCR, shSET8, shMTA1, or shSET8 in combination with shMTA1) by RT-qPCR and western blot

To further investigate the functional implications of the physical association between SET8 and MTA1/NuRD complex, and to explore the biological significance, we proceeded to investigate the genome-wide transcriptional targets of SET8 and MTA1 using chromatin IP-based deep sequencing (ChIP-seq) in MIA Paca-2 cells. In these experiments, ChIP assays were conducted, SET8 and MTA1 associated DNAs were amplified respectively. Subsequently, the amplified DNA fragments were subjected to unbiased labeling and sequencing using the HiSeq2500 platform. The resultant analysis revealed the genomic distribution of relative peaks, such as promoters, 5’ untranslated regions (UTR5), 3’ untranslated regions (UTR3), exons, introns, downstream regions (≤3 kb), and distal intergenic regions (Fig. 4A). Furthermore, the peaks’ chromosome distribution is shown in Fig. 4B. We employed DREME (the Discriminative Regular Expression Motif Elicitation) and identified a shared binding motif for MTA1 and SET8 (Fig. 4C), supporting the physical interaction as well as the functional connection between SET8 and MTA1. We identified 1409 SET8 specific binding peaks and 1385 MTA1 specific binding peaks in the promoter region. The data were then cross-analyzed to identify overlapping DNA sequences/gene promoters between SET8 and MTA1. Consequently, a total of 149 distinct promoters were considered as targets of the SET8/MTA1/NuRD complex using Venn diagram (Fig. 4D). The genes regulated by these promoters were analyzed through gene ontology using the DAVID bioinformatics resource. GO enrichment analysis revealed significant terms related to cell migration, positive regulation of the MAPK cascade, and microtubule cytoskeleton organization—processes that are all closely linked to angiogenesis (Fig. 4E). To validate the findings, qChIP analyses were conducted in MIA Paca-2 cells using specific antibodies against SET8 or MTA1 on selected genes such as *SOCS2*, *BATF2*, *TRIM22*, *FDX1*, *PARP11*, *USP10*, *PEX3*, *IGFBP6*. These analyses revealed strong enrichment of SET8 and MTA1 on the promoters of these genes, which further validating the ChIP-seq results. GAPDH was used as a negative control, and similar trends were observed in qChIP analyses conducted in AsPC-1 cells (Fig. 4F). In addition, qChIP analysis with specific antibodies against H4K20me1, a catalytic product of SET8, demonstrated the presence of H4K20me1 marks on the target promoters, thus corroborating the association of SET8 with these genomic loci (Fig. 4G). Importantly, qChIP experiments revealed that depletion of SET8 or MTA1, not only caused remarkable reduction in the recruitment of the respective proteins at the target gene promoters, but also reduced the recruitment of the other factor of the SET8/MTA1 complex. Notably, the levels of H4K20me1 were diminished, while the levels of H3ac exhibited a marked increase at the representative target promoters following the knockdown of either SET8 or MTA1 (Fig. 4H), indicating that SET8 and MTA1/NuRD are closely connected in mediating transcriptional repression, with H3 ChIP was used as control. Therefore, we further investigated whether SOCS2 serves as a potential target regulated by SET8/NuRD (MTA1) complex. For this purpose, MIA Paca-2 or AsPC-1 cells were transfected with either control shSCR (scrambled shRNA) or shRNAs specifically targeting SET8 or MTA1 (referred to hereafter as shSET8 and shMTA1). Analysis by RT-qPCR showed that knockdown of either SET8 or MTA1 increased the expression of SOCS2. Consistently, the protein levels of SOCS2 exhibited an increase as measured by western blot, and co-knockdown of SET8 and MTA1 led to more pronounced changes in the expression of SOCS2 in both MIA Paca-2 cells and AsPC-1 cells (Fig. 4I–J). Taken together, these results demonstrated that SET8 and subunits of the MTA1/NuRD complex are physically associated and functionally connected in one multiunit complex to repress downstream target genes.

### SOCS2 inhibits invasion and angiogenic potential of PDAC cells

**Fig. 5:**
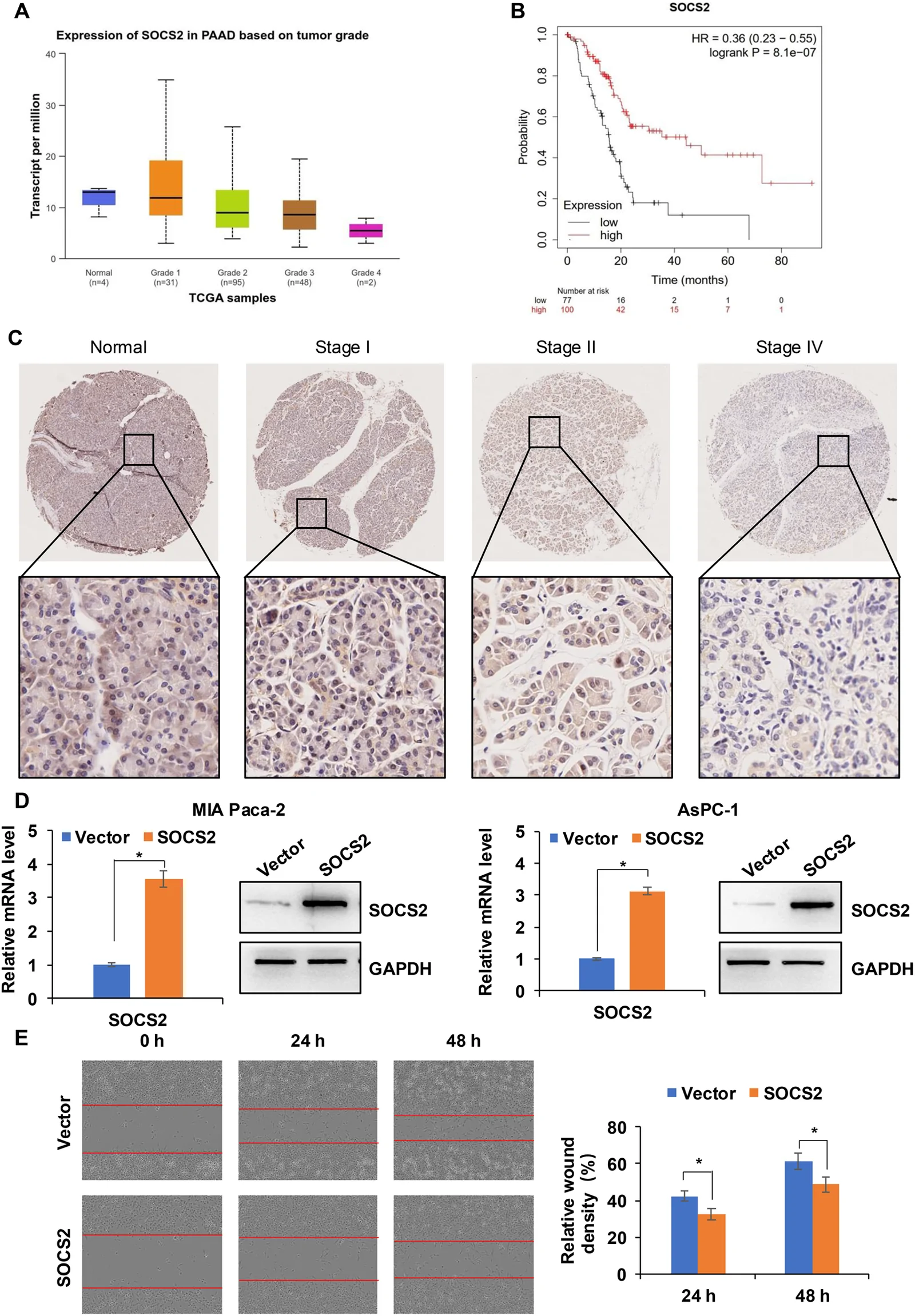

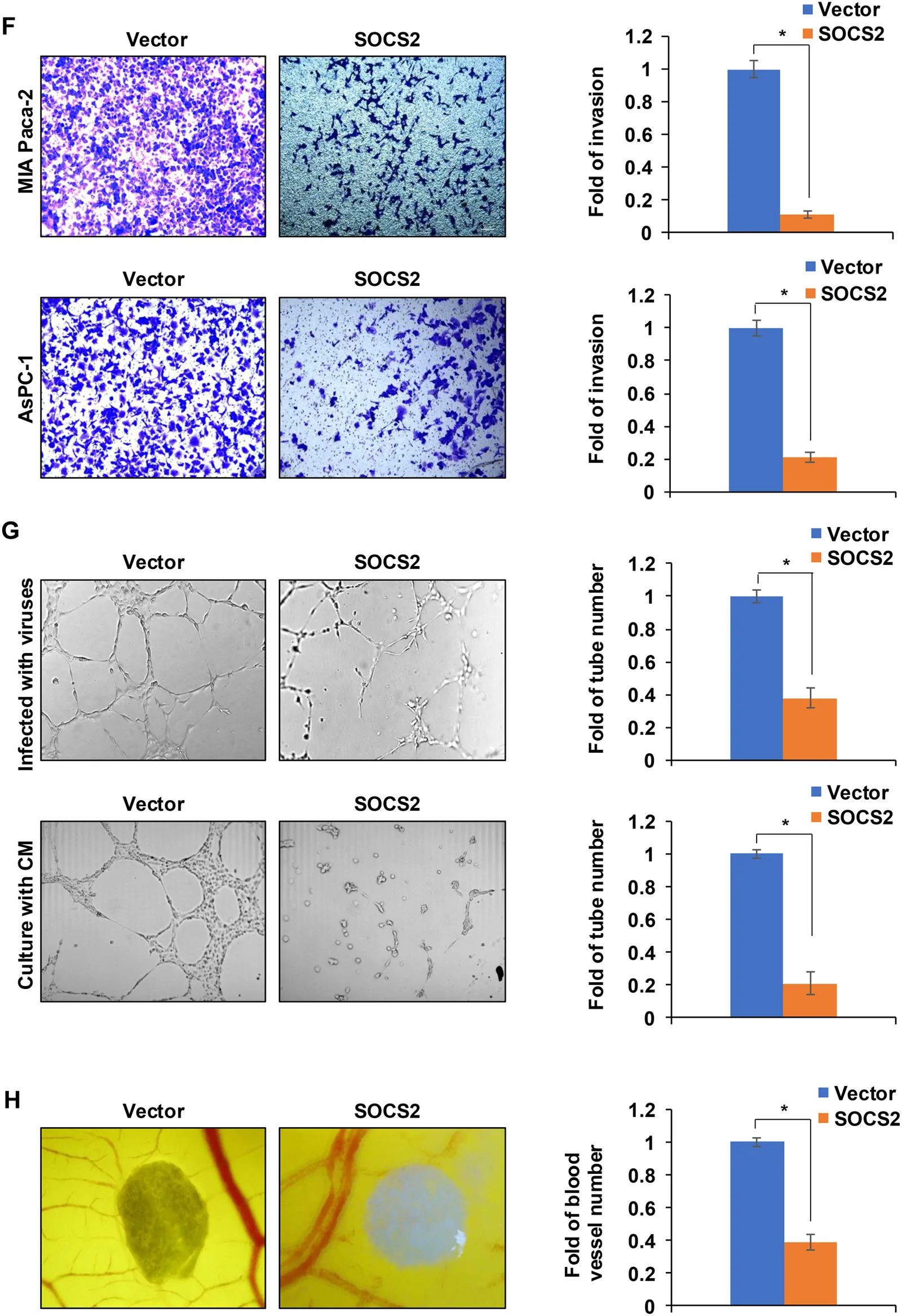
SOCS2 inhibits invasion and angiogenic potential of PDAC cells. (**A**) The expression levels of SOCS2 were assessed in Grade1-4 PDAC tissues compared to normal tissues using the UALCAN datasets. (**B**) The correlation between SOCS2 expression and survival times in PDAC was analyzed utilizing the online analysis tool https://www.kmplot.com/analysis. (**C**) Immunohistochemistry staining images targeting SOCS2 (1:100, YM960233, Immunoway) were obtained from PDAC specimens (*n* = 72) and adjacent para-carcinoma tissues (*n* = 74). Scale bars: up panel, 200 μm; Down panel, 40 μm. (**D**) MIA Paca-2 or AsPC-1 cells were infected with specific lentivirus expressed Vector or SOCS2 overexpression construction. Subsequently, the mRNA and protein levels of SOCS2 were quantified. The migratory capacity was evaluated by (**E**) wound healing and (**F**) transwell assays treated with Vector or SOCS2, with representative images presented. HUVECs were infected with (**G**) lentiviruses carrying vector or SOCS2 lentiviruses, or cultured with conditioned media from MIA Paca-2 cells infected with lentiviruses carrying vector or SOCS2 and were added onto a solidified extracellular matrix. After incubation, the number of endothelial tubes formed was counted under a light microscope, with representative images from each group are shown. Scale bars: 100 μm. (**H**) MIA Paca-2 cells were infected with viruses carrying the indicated constructs for YSM analysis. The number of blood vessels that migrated into the sponge was counted and representative images from each group were provided. Each bar represents the mean±SD. Each independent experiment was performed at least three times

Among the identified common target genes *SOCS2* might be a potential tumor suppressor in PDAC. As documented by *MingJie He* et al., SOCS2 exhibited significant downregulation in pancreatic cancer cells, and the enforced expression of SOCS2 notably reduced the viability of PANC-1 and Capan-2 cells [20]. Despite these observations, the molecular mechanisms and functional roles of SOCS2 in PDAC remain elusive. Using UALCAN datasets, we substantiated that the expression levels of SOCS2 were diminished in PDAC tissues compared with normal pancreatic tissues, displaying a negative correlation with histological grade (Fig. 5A). Moreover, elevated SOCS2 is associated with favorable outcome (Fig. 5B). Through immunohistochemical staining of PDAC TMA samples, a statistically significant reduction in SOCS2 expression was observed in carcinomas compared to that in the para-carcinoma tissue, and its expression was negative correlated with more advanced AJCC stages (Fig. 5C). To investigate the functional role of SOCS2 in PDAC, SOCS2 construct was transducted in MIA Paca-2 cells or AsPC-1 cells. The efficacy of overexpression was evaluated through both RT-qPCR and western blot analyses. The findings demonstrated a significant increase in SOCS2 expression following transfection (Fig. 5D). Subsequently, the monolayer wound healing assay was used to assess cell migration. Notably, the overexpression of SOCS2 showed a significant deceleration in closure of the wound scratch width after 24 and 48 hours compared with the vector groups (Fig. 5E). Moreover, the Matrigel invasion assay revealed that the percentages of migrated MIA Paca-2 cells or AsPC-1 cells through the polycarbonate membrane in the SOCS2 overexpression group were significantly reduced compared to the control groups (Fig. 5F). As angiogenesis is closely related to tumor invasion and metastasis in PDAC [21], to test that whether SOCS2 could impact angiogenesis in PDAC, initially, we conducted in vitro endothelial tube formation assays to assess the ability of endothelial cells to form three-dimensional capillary-like tubular structures. Human umbilical vein endothelial cells (HUVECs) or MIA Paca-2 cells were transduced with lentiviruses carrying vector or SOCS2 construct. Conditional media (CM) from MIA Paca-2 cells were added onto solidified extracellular matrix gels. After incubation, the formation of endothelial cell tubes was examined using a light microscope, and the number of tubes was counted. The result revealed that SOCS2 overexpression HUVECs or HUVECs cultured in CM from SOCS2 overexpression MIA Paca-2 cells exhibited a reduced formation of endothelial tubes compared to the vector control groups, suggesting that SOCS2 is capable of inhibiting tube forming potential (Fig. 5G). Furthermore, In vivo chicken yolk sac membrane (YSM) assays were then carried out in which gelatin sponges that had adsorbed suspensions of MIA Paca-2 cells infected with viruses carrying vector or SOCS2 were placed on top of the YSM. Microscopic analysis of the number of blood vessels entering the sponge showed that, in comparison to the control group, eggs treated with SOCS2 overexpression MIA Paca-2 cells formed significantly fewer blood vessels (Fig. 5H). The results provide evidence supporting the involvement of SOCS2 in suppressing blood vessel.

### Functional interdependence of SET8 and MTA1 in enhancing the invasion and angiogenic potential of PDAC cells

**Fig. 6:**
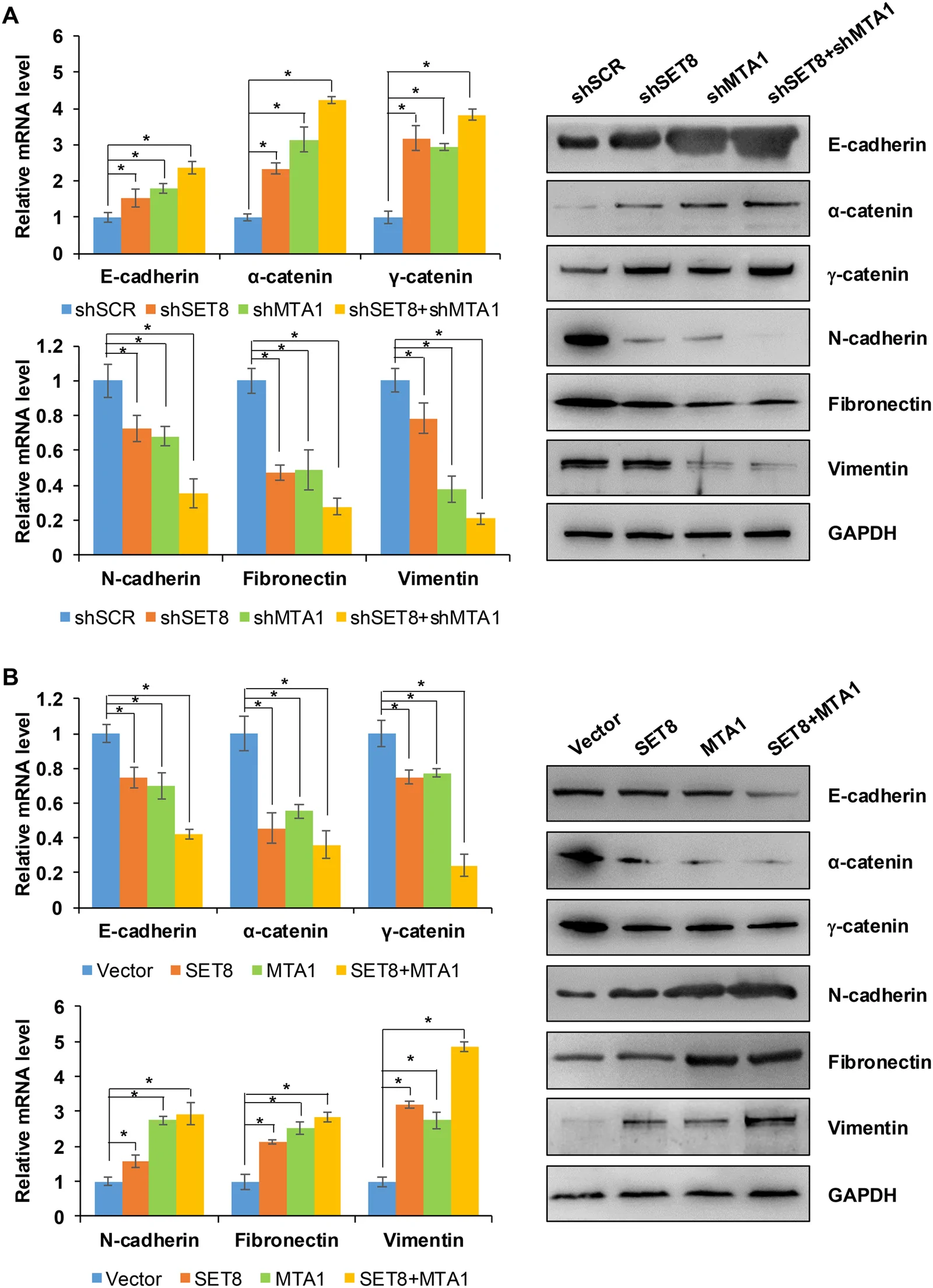

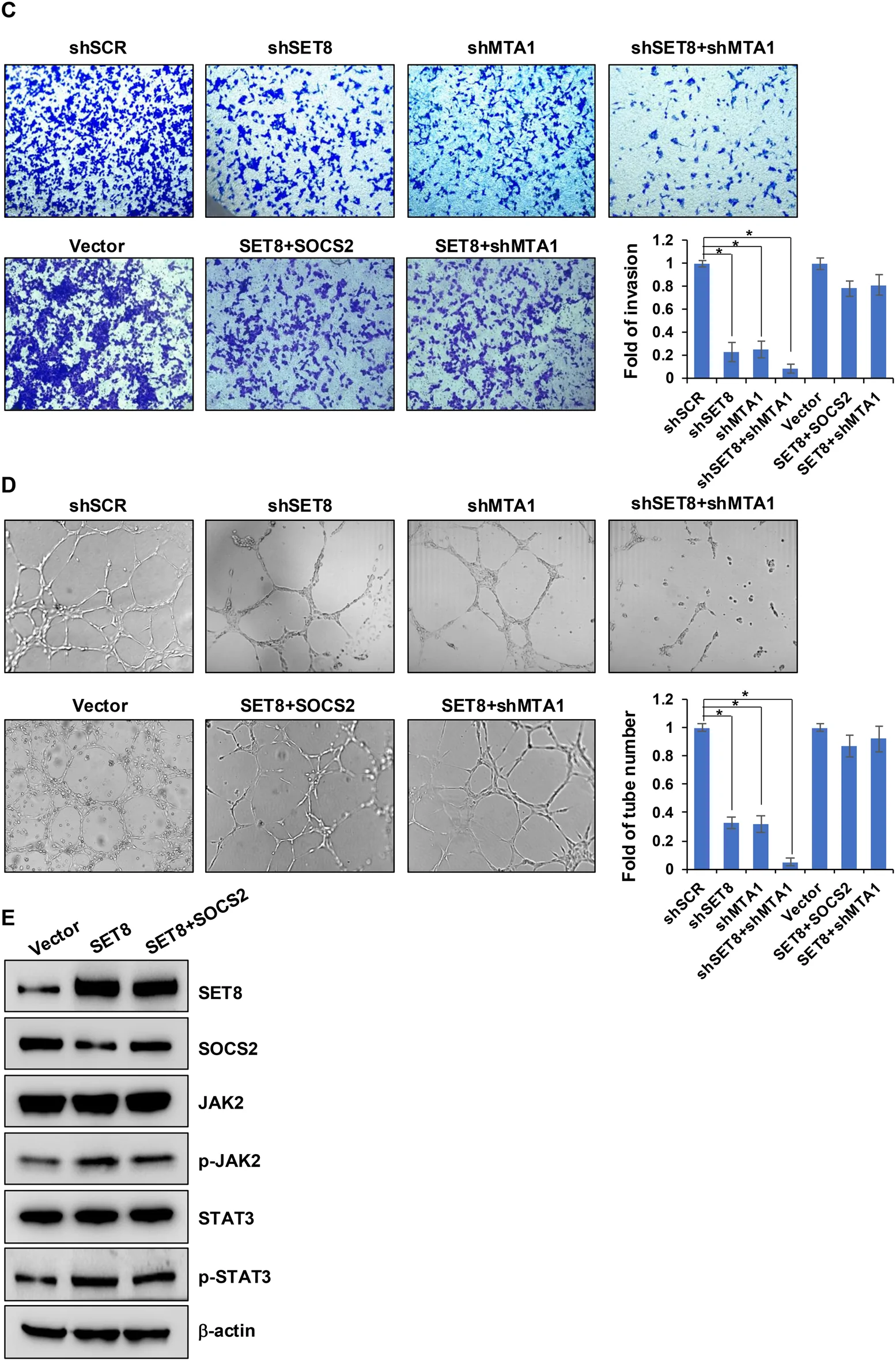
Functional interdependence of SET8 and MTA1 in enhancing the invasion and angiogenic potential of PDAC cells. The expression levels of specified epithelial or mesenchymal markers were measured using RT-qPCR or westernblot in MIA Paca-2 cells infected with lentiviruses carrying (**A**) shSCR, shSET8, shMTA1 or shSET8 combined with shMTA1, (**B**) Vector, SET8, MTA1 or SET8 combined with MTA1 respectively, at the indicated time points. Error bars represent the mean ± SD derived from three distinct experiments. (**C**) MIA Paca-2 cells were infected with lentiviruses containing specific shRNA or designated expression constructs. The invaded cells were stained, counted under the microscope and images represent a single field of view per group. Scale bars: 100 μm. (**D**) HUVECs were cultured with CM from MIA Paca-2 cells infected with retroviruses carrying control shRNA, shSET8 or shMTA1, shSET8 and shMTA1, SET8 and SOCS2, or SET8 and shMTA1, and then added onto a solidified extracellular matrix. After incubation, endothelial cell tube formation was assessed and the tube number was counted under light microscopy. Representative images are shown. Error bars represent the mean±SD for triplicate experiments. (**E**) Assessment of total JAK2, STAT3, phosphorylation JAK2 and STAT3 expression via western blot analyses after SET8 overexpression and treatment with SOCS2

The identification of SOCS2 as target of the SET8/MTA1/NuRD complex, and the role of SOCS2 as a tumor suppressor in the development and progression of PDAC. Subsequently, we investigated the potential involvement of SET8/MTA1/NuRD complex in carcinogenesis. For this purpose, loss -/gain -of function of SET8 and/or MTA1 was carried out in MIA Paca-2 cells. The expression of epithelial/mesenchymal markers was analyzed. Our investigations revealed that knockdown of either SET8 or MTA1 resulted in the upregulation, at both mRNA and protein levels, of epithelial protein markers including E-cadherin, α-catenin as well as γ-catenin. Conversely, the expression of mesenchymal markers, including N-cadherin, fibronectin, and vimentin, were downregulated, and the simultaneous knockdown of SET8 and MTA1 resulted in more pronounced alterations in the expression of these markers (Fig. 6A). Conversely, the overexpression of SET8, MTA1, or their combination resulted in the reduction of epithelial markers and induction of mesenchymal markers (Fig. 6B). Cell invasion capabilities were assessed by transwell assays, revealing that compared to the control, SET8 or MTA1 knockdown significantly inhibited the invasion of PDAC cells, and in combination depletion of SET8 and MTA1 led to accumulation effect. Notably, trigger effect of SET8 in invasion appeared, at least in part, to be through the repression of SOCS2, as simultaneous overexpression of SOCS2 could rescue the invasive phenotype by SET8-overexpressed models. Interestingly, the effect of SET8 overexpression that was probably mediated by its interaction with the NuRD complex, which could be rescued by simultaneous inhibition of MTA1 (Fig. 6C). Given the established role of SOCS2 in angiogenesis, it is reasonable to hypothesize that SET8/MTA1 may play a role in the regulation of angiogenesis. To investigate this hypothesis, an in vitro endothelial tube formation assay was performed, we found that knockdown of SET8 or MTA1 was correlated with a reduction in the tube-forming capacity of HUVECs cells, with a more pronounced decrease observed with combined knockdown of SET8 and MTA1. Furthermore, restoration of SOCS2 significantly attenuated the oncogenic effects induced by SET8 overexpression, supporting the presence of a functional relationship between SET8 and SOCS2. The effect of SET8 on the invasiveness of PDAC seemed to be mediated through cooperative interactions with the NuRD complex, as evidenced by the diminished impact of SET8 overexpression when MTA1 was concurrently inhibition (Fig. 6D). Considering SOCS2 is a key negative regulator of the Janus kinase (JAK)-Signal Transducers and Activators of Transcription (STAT) signaling cascade [22, 23], we next examined this pathway. Western blot results demonstrated that SOCS2 expression in the context of SET8 overexpression inhibited the phosphorylation of JAK2 and STAT3, leading to a blockade of core pathway signaling (Fig. 6E).

### SET8 cooperates with MTA1 to promote PDAC carcinogenesis through SOCS2

**Fig. 7:**
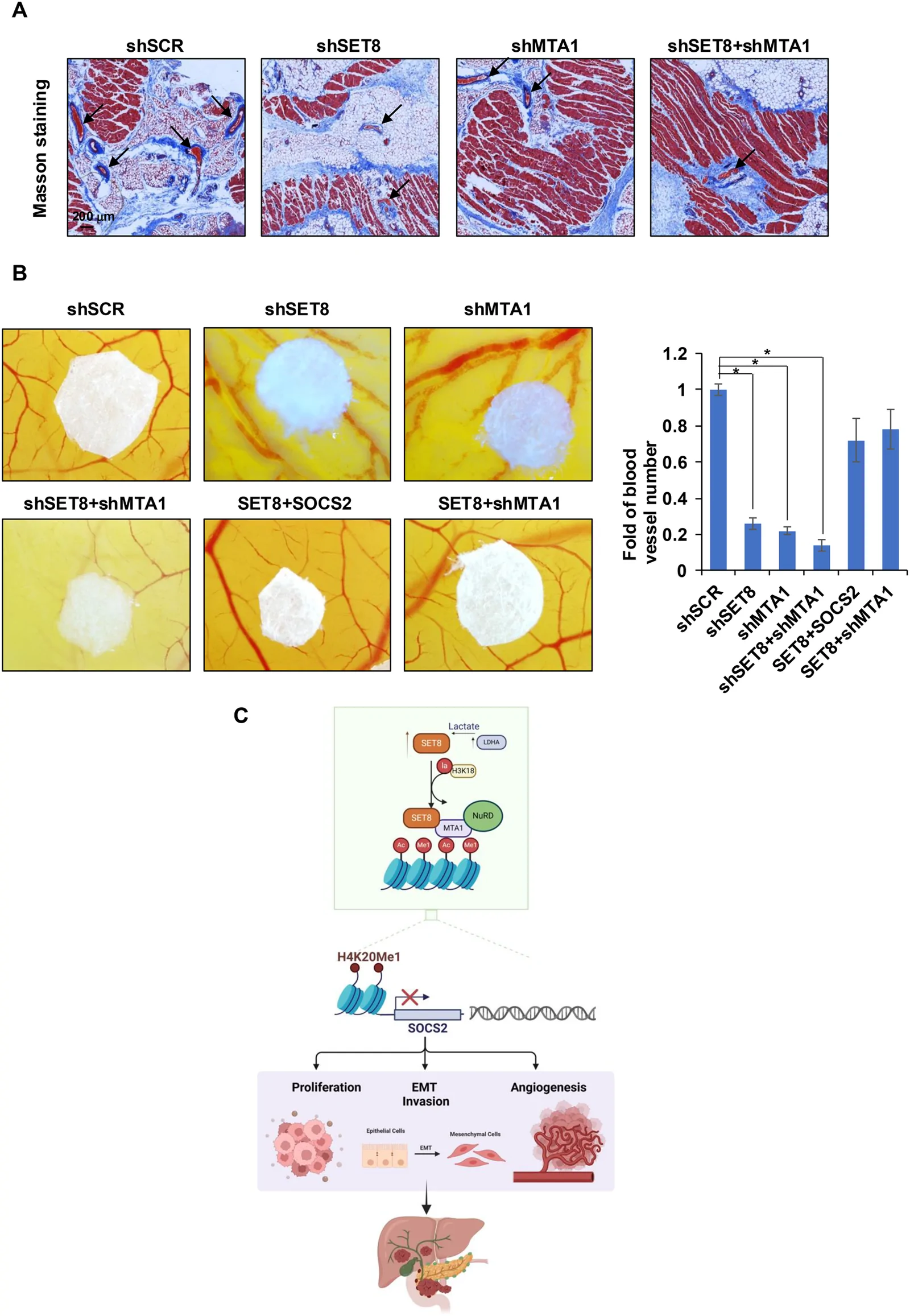
SET8 cooperates with MTA1 to promotes PDAC carcinogenesis through SOCS2. (**A**) Matrigel mixed with MIA Paca-2 cells infected with lentiviruses carrying the indicated specific shRNA and/or expression constructs were subcutaneously implanted into the left underarm BALB/c nude mice (*n* = 6). Seven days after injection, the mice were sacrificed, and the Matrigel plugs were removed and stained with Masson trichrome. Scale bar = 200 μm. Representative images are presented and quantification. Error bars represent mean ± SD for three independent measurements. (**B**) MIA Paca-2 cells were infected with viruses carrying indicated constructs for YSM analysis. The number of blood vessels that migrated into the sponge was counted. Representative images from each group are displayed. Each bar represents the mean ± SD for triplicate measurements, *p < 0.05. Scale bar = 200 μm. (**C**) Proposed model of the SET8 in PDAC carcinogenesis

To investigate the role of SET8 in PDAC carcinogenesis in vivo, a Matrigel plugs assay was conducted. Immunodeficient nude mice were injected with Matrigel containing MIA Paca-2 cells infected with control lentivirus shRNA vector, shSET8 or/and shMTA1. These cells were subsequently implanted into the left underarm of immunodeficient nude mice. Microscopic examination of Masson’s trichrome-stained Matrigel plugs revealed that the number of vessel-like structures (defined as lumen-containing red blood cell clusters surrounded by collagen-rich stroma) was significantly reduced in the SET8 or MTA1 knockdown groups compared to the control group. The combined knockdown of SET8 and MTA1 resulted in a more pronounced reduction in vascular structure formation (Fig. 7A).

Next, in vivo YSM assay was carried out, in which gelatin sponges adsorbed suspensions of MIA Paca-2 cells transfected with shSET8, and/or shMTA1 or shSCR were utilized. In the eggs treated with SET8 knockdown MIA Paca-2 cells developed less blood vessels toward the implants, displaying a characteristic “spoked wheel” pattern. Consistent with the established functional relationship between SET8/MTA1/NuRD and SOCS2. Furthermore, the effect of SET8 on the angiogenesis potential of PDAC could be restored by knockdown MTA1 (Fig. 7B). Taken together, these results support a role for histone lactylation induced SET8 expression in modulating the invasive and angiogenic potential of PDAC cells. This regulatory function is likely through the interaction of SET8 with the MTA1/NuRD complex, leading to the suppression of target genes including SOCS2 (Fig. 7C).

## Discussion

The precise mechanism by which SET8 modulates the progression of PDAC has not been clearly elucidated. Through immunohistochemical and western blot analyses, a notable elevation in SET8 expression was observed in PDAC patients. Moreover, the Kaplan‑Meier plot showed that higher expression of SET8 was correlated with lower overall survival times among PDAC patients. Our investigations revealed that not only knockdown of LDHA but also different kinds of glycolysis inhibitors could attenuate SET8 expression. Moreover, a positive correlation between histone lactylation and SET8 expression was established, which indicates SET8 might be a potential lactylation driven gene and a promising therapeutic target for PDAC treatment.

Numerous investigations have indicated that SET8 exerts its biological functions through interaction with a various of nuclear proteins, such as proliferating cell nuclear antigen (PCNA), TWIST, RNA polymerase II, and ER [24]. In our current study, the likelihood of this physical association between SET8 and the MTA1/NuRD complex is bolstered by results from several molecular experiments, such as affinity purification, co-IP. Significantly, the physical association between SET8 and MTA1/NuRD provides a plausible molecular mechanism for biochemically repression a panel of genes, including SOCS2, which is a potent suppressor of PDAC progression.

In agreement with our findings, recent studies have also highlighted SET8 functions as an oncogene in several types of cancers. For example, in breast cancer, SET8 expression has been positively linked to epithelial–mesenchymal transition, thereby enhancing the invasive potential of breast cancer cells both in vitro *and* in vivo [25]. In hepatocellular carcinoma, depletion of SET8 can inhibit the proliferation and invasion while increasing sensitivity to chemotherapy [16]. Histone methylation status in different lysine residues plays distinct roles in transcriptional regulation. Interestingly, Histone H4K20 monomethylation by SET8 has been reported to be involved in both transcriptional activation and repression. As recruited by LEF1/TCF4, SET8 is crucial for activation of Wnt signaling target genes [26].

The NuRD complex is a chromatin-associated protein complex which contains two major enzymatic functions, chromatin remodeling and histone deacetylase activity [27]. This complex can assume multiple mutually exclusive conformations and is involved in various cellular functions, such as somatic reprogramming [28], cell lineage differentiation [29], DNA damage response [30], aging [31] and carcinogenesis [32]. In addition, the NuRD complex has been shown to interact with numerous proteins such as GATA3, ZEB2 [17], Snail [33], RUNX2 [34] and LHX2 [35], some of which act as recruiters for NuRD to specific target sites. Moreover, the other proteins adding new histone demethylation or methylation activity to this complex such as PRMT5 [33], JARID2 [36] and G9A [17], suggesting ongoing efforts to identify NuRD-interacting proteins. In our current investigation, we have validated that SET8 recruits the NuRD complex to form a transcriptional inhibitory unit and adds histone H4K20 monomethylation activity to the complex. Although our current study focused on the pro-angiogenic role of the axis, the SET8/MTA1/NuRD complex may also modulate immune cell recruitment or function—an important aspect that warrants further investigation in subsequent studies.

In our previous study, we initially elucidated the mechanism underlying elevated MTA2 expression. As is known, NuRD complexes contain several core subunits such as CHD3, CHD4, MTA1/2/3, HDAC1, HDAC2, whose pattern of expression is heterogeneous across different tissues and various cell types. Especially, members of the MTA family do not co-localize in the same NuRD complex and are not functionally substitute for each other [17]. In our current research, we have highlighted MTA1 as another critical factor associated with invasion in PDAC cells. The novel epigenetic signaling pathway provides evidence for MTA1 as a promising clinical therapeutic target in PDAC. More importantly, beyond cell invasion, angiogenesis plays a critical role in tumor growth and metastasis by facilitating nutrient and oxygen supply [37].

Epigenetics modification has been found play key roles in pancreatic cancer [38]. As reported, histone lactylation was elevated in PDAC, and the high level of H3K18la was associated with poor prognosis. The suppression of glycolytic activity by LDHA knockdown contributed to the anti-tumor effects of PDAC in vitro and in vivo. Our results demonstrate for the first time that knocking down LDHA suppresses SET8 expression by modulating H3K18 lactylation, thereby elucidating the molecular mechanism through which it inhibits PDAC progression. This provides new perspectives and additional evidence supporting the targeting of LDHA as a potential therapeutic strategy.

Considering the relationship between LDHA and SET8, it is reasonable to hypothesize that SET8 serves as a potent oncogene in PDAC. Our study demonstrates that the SET8/MTA1/NuRD complex promotes the invasion and tube formation of PDAC cells in vitro, and promotes the carcinogenesis and angiogenesis of PDAC in vivo. This complex exerts its effects in a coordinated and cooperative manner to suppress the expression of target genes. As reported, polymorphisms are essential in determining the effectiveness and outcome of treatments in Multiple Myeloma [39]. Meta-analytical and meta-regression evaluations [40] may be applied in future research to examine SET8 polymorphism in PDAC, thus enabling an in-depth exploration of the molecular mechanisms.

The suppressor of cytokine signaling 2 (SOCS2) has been identified to act as a tumor suppressor in breast cancer progression, prostate cancer, gastric cancer [23, 41, 42]. As He, M et al. reported, overexpression of SOCS2 significantly decreased the viability of PANC-1 and Capan-2 cells, decreased mitochondrial DNA content and ATP levels, and caused mitochondrial characterized by an accumulation of reactive oxygen species (ROS) [20]. In our investigation, we have demonstrated that downregulation of SOCS2 is modulated by SET8/MTA1/NuRD complex, which expands the current understandings of SOCS2 serving as a critical tumor suppressor in PDAC. While SOCS2 served as a functionally validated key target, the other 148 common target genes identified may collectively contribute to the phenotypic outcomes through coordinated regulation of related pathways (e.g., hypoxia response, metabolic adaptation). Our clinical validation was performed in a Chinese patient cohort, and therefore caution should be exercised when extrapolating the results to other ethnic populations. Multi-center, multi-ethnic studies will be needed to confirm the broader applicability of our finding.

Our findings not only provide a molecular basis for the action between histone lactylation and SET8 in PDAC progression, but also contribute to elucidating the intricate molecular interplays within the regulatory network of the NuRD complex. Targeting the lactylation-SET8/MTA1/NuRD axis represents a promising therapeutic strategy for PDAC, potentially employing agents such as the LDHA inhibitor FX11, SET8-specific inhibitors, or MTA1 disruptors. Furthermore, these findings could facilitate the design of prognostic tools, addressing a critical unmet need in PDAC management.

## Electronic supplementary material

Below is the link to the electronic supplementary material.

Supplementary Material 1

Supplementary Material 2

Supplementary Material 3

## Data Availability

The analyzed data sets generated during the study are available from the corresponding author, on reasonable request.
