# Supplementary material for: Histone lactylation boosted SET8 potentiates carcinogenesis and angiogenesis of pancreatic ductal adenocarcinoma by cooperating with MTA1/NuRD complex
Source: J Transl Med. 2026 Jun 26;24:1181. doi: 10.1186/s12967-026-08424-3 (PMC13576197; doi:10.1186/s12967-026-08424-3)
Supplement: Supplementary file 1 [file 12967_2026_8424_MOESM2_ESM.docx]

| Primer | Sequences (5'-3') |
| --- | --- |
| SET8 forward primer | GGTGACAGAGTGGAGCCAT |
| SET8 reverse primer | GTAAATACGTTCTCCCCGTCG |
| LDHA forward primer | ATGGCAACTCTAAAGGATCAGC |
| LDHA reverse primer | CCAACCCCAACAACTGTAATCT |
| E-cadherin forward primer | CGAGAGCTACACGTTCACGG |
| E-cadherin reverse primer | GGGTGTCGAGGGAAAAATAGG |
| α-catenin forward primer | GGGGATAAAATTGCGAAGGAGA |
| α -catenin reverse primer | GTTGCCTCGCTTCACAGAAGA |
| γ-catenin forward primer | TCTCCAACCTGACATGCAACA |
| γ-catenin reverse primer | CATAGTTGAGACGCACAGAGTTC |
| N-cadherin forward primer | AGCCAACCTTAACTGAGGAGT |
| N-cadherin reverse primer | GGCAAGTTGATTGGAGGGATG |
| Fibronectin forward primer | CGGTGGCTGTCAGTCAAAG |
| Fibronectin reverse primer | AAACCTCGGCTTCCTCCATAA |
| Vimentin forward primer | GACGCCATCAACACCGAGTT |
| Vimentin reverse primer | CTTTGTCGTTGGTTAGCTGGT |
| GAPDH forward primer | AGGTCGGAGTCAACGGATTT |
| GAPDH reverse primer | TGGAATTTGCCATGGGTGGA |

Supplementary Table 1. RT-qPCR Primer sequence
