# Supplementary material for: Histone lactylation boosted SET8 potentiates carcinogenesis and angiogenesis of pancreatic ductal adenocarcinoma by cooperating with MTA1/NuRD complex
Source: J Transl Med. 2026 Jun 26;24:1181. doi: 10.1186/s12967-026-08424-3 (PMC13576197; doi:10.1186/s12967-026-08424-3)
Supplement: Supplementary file 2 [file 12967_2026_8424_MOESM4_ESM.docx]

Supplementary Table S2. Clinic pathologic variables in TMA PDAC patients (n=79)

| Variables | No. (n=79) | SET8 expression | | *P* value |
| --- | --- | --- | --- | --- |
|  |  | Low (n=36) | High (n=43) |  |
| Age |  |  |  |  |
| < 50 | 10 | 6 | 4 | 0.327 |
| ≥ 50 | 69 | 30 | 39 |  |
| Gender |  |  |  |  |
| Male | 47 | 20 | 27 | 0.514 |
| Female | 32 | 16 | 16 |  |
| Tumor size † |  |  |  |  |
| Small (< 5 cm) | 59 | 29 | 30 | 0.012 |
| Large (≥ 5 cm) | 15 | 2 | 13 |  |
| Pathological grading |  |  |  |  |
| I-II | 40 | 23 | 17 | 0.031 |
| III | 39 | 13 | 26 |  |

†Tumor size data were unavailable for 5 of 79 patients (6.3%). Percentages and statistical analyses are based on available cases (n=74).
